# Prediction of drug-induced nephrotoxicity and injury mechanisms with human induced pluripotent stem cell-derived cells and machine learning methods

**DOI:** 10.1038/srep12337

**Published:** 2015-07-27

**Authors:** Karthikeyan Kandasamy, Jacqueline Kai Chin Chuah, Ran Su, Peng Huang, Kim Guan Eng, Sijing Xiong, Yao Li, Chun Siang Chia, Lit-Hsin Loo, Daniele Zink

**Affiliations:** 1 Institute of Bioengineering and Nanotechnology, 31 Biopolis Way, The Nanos, Singapore 138669, Singapore; 2 Bioinformatics Institute, 30 Biopolis Street, #07-01 Matrix, Singapore 138671, Singapore; 3 Department of Pharmacology, Yong Loo Lin School of Medicine, National University of Singapore, 10 Medical Drive, Singapore 117597, Singapore

## Abstract

The renal proximal tubule is a main target for drug-induced toxicity. The prediction of proximal tubular toxicity during drug development remains difficult. Any *in vitro* methods based on induced pluripotent stem cell-derived renal cells had not been developed, so far. Here, we developed a rapid 1-step protocol for the differentiation of human induced pluripotent stem cells (hiPSC) into proximal tubular-like cells. These proximal tubular-like cells had a purity of >90% after 8 days of differentiation and could be directly applied for compound screening. The nephrotoxicity prediction performance of the cells was determined by evaluating their responses to 30 compounds. The results were automatically determined using a machine learning algorithm called random forest. In this way, proximal tubular toxicity in humans could be predicted with 99.8% training accuracy and 87.0% test accuracy. Further, we studied the underlying mechanisms of injury and drug-induced cellular pathways in these hiPSC-derived renal cells, and the results were in agreement with human and animal data. Our methods will enable the development of personalized or disease-specific hiPSC-based renal *in vitro* models for compound screening and nephrotoxicity prediction.

The kidney is a main target for drug-induced toxicity. The renal proximal tubular cells (PTC) are frequently affected due to their roles in glomerular filtrate concentration and drug transport^1^,^2^. Many widely used marketed drugs including anti-cancer drugs, antibiotics, immunosupressants and radiocontrast agents are nephrotoxic and injure PTC^2^,^3^. Drug-induced nephrotoxicity can lead to acute kidney injury (AKI) or chronic kidney disease in patients and is a major problem for clinicians^2^,^3^. Development of less nephrotoxic drugs is challenging due to the fact that the prediction of nephrotoxicity during drug development remains difficult. Typically, compound nephrotoxicity is only detected during late stages of drug development, which is associated with high costs for the pharmaceutical industry^4^. Animal models have limited predictivity and the development of renal *in vitro* models with high predictivity has been challenging^1^,^2^.

Recently, we have established a cell-based *in vitro* model that predicts PTC-toxicity in humans with high accuracy^5^. This model used increased expression of interleukin (IL)6 and IL8 as endpoint, and employed human primary renal proximal tubular cells (HPTC). Due to various issues associated with primary cells (cell sourcing problems, inter-donor variability, functional changes during passaging) stem cell-based approaches would be preferred.

By using human embryonic stem cells (hESC) we have developed the first protocol that allows to differentiate stem cells into HPTC-like cells^6^. Applying such hESC-derived cells in the IL6/IL8-based *in vitro* model allowed identification of compounds that injure the proximal tubule in humans^7^. However, use of hESC-derived HPTC-like cells resulted in relatively low sensitivity compared to HPTC. Also, the differentiation period comprised 20 days when the hESC-based approach was used, which made this model relatively inefficient. Further, due to ethical and legal issues associated with hESC, hESC-based assays for drug safety screening are not widely applicable. Also, it would be difficult to establish patient-specific HPTC-like cells and personalized models with hESC-based approaches.

In order to address these issues it is necessary to develop renal *in vitro* models based on HPTC-like cells derived from human induced pluripotent stem cells (hiPSC). Further, it would be most desirable if hiPSC-derived HPTC-like cells could not only be used for the prediction of drug-induced nephrotoxicity, but also for the identification of underlying injury mechanisms and drug-induced cellular pathways. In addition, hiPSC-derived renal cell-based models should be suitable for automated cellular imaging in order to allow efficient analysis of larger numbers of compounds. Currently no renal *in vitro* model is available that would be suitable for automated cellular imaging. Furthermore, no *in vitro* model based on hiPSC-derived renal cells is available, neither for the prediction of nephrotoxicity, nor for the analysis of cellular pathways and injury mechanisms.

Recently, a variety of protocols have been developed for the differentiation of human or murine embryonic (ESC) or induced pluripotent stem cells (iPSC) into the renal lineage^8^,^9^,^10^,^11^,^12^,^13^. These protocols were designed to recapitulate embryonic kidney development and involved multiple steps to mimic the different stages. The main goal of these approaches, which typically generated kidney precursors and a mix of different renal cell types, were applications in disease models and regenerative medicine. Any application or *in vitro* model based on these protocols has not been developed, so far.

Here, we report a rapid and simple 1-step protocol for the differentiation of hiPSC into HPTC-like cells with >90% purity. Using this protocol, compound screening could be immediately performed after a differentiation period of only 8 days without the requirement of cell harvesting or purification. The combination of the hiPSC-based renal *in vitro* model with machine learning methods allowed us to predict drug-induced proximal tubular toxicity in humans with high accuracy. Injury mechanisms and drug-induced cellular pathways could be reliably identified by using automated cellular imaging.

## Results and Discussion

### Differentiation of hiPSC into HPTC-like cells

**Figure 1:**
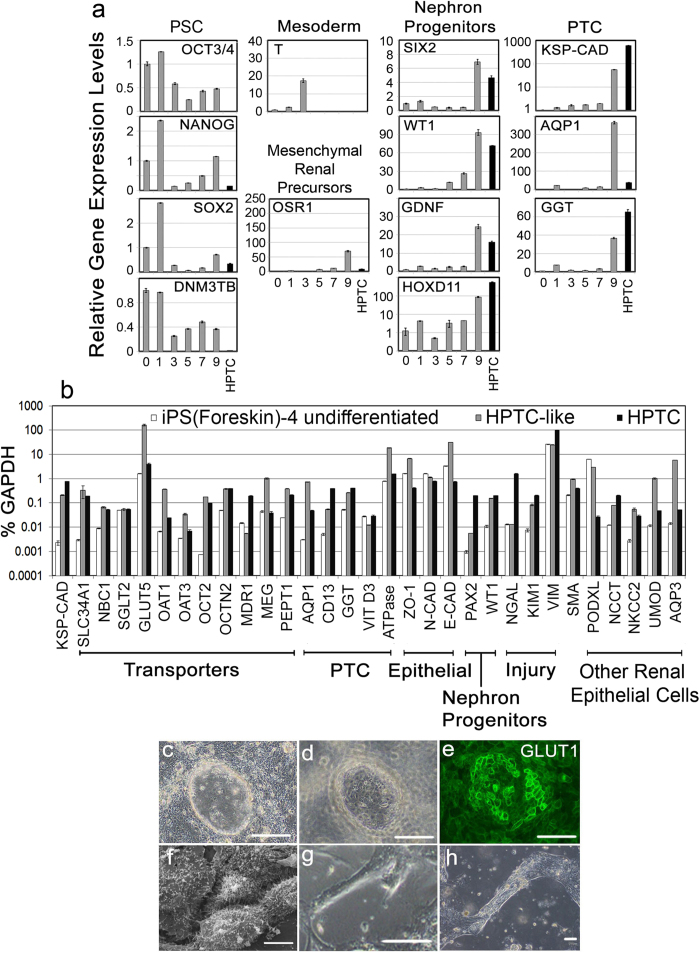
Marker expression and morphology of iPS(Foreskin)-4-derived cells (**a**) Marker expression during differentiation. Marker expression levels were determined by qPCR on every other day during a time period of 9 days (gray bars). The differentiation protocol was applied on day 1. The bars show the mean +/− standard deviation (s.d., n = 3). Marker expression levels were normalized to the expression levels of the respective marker in undifferentiated iPS(Foreskin)-4 cells (day 0). Mean expression levels in undifferentiated hiPSC were set to 1. Black bars indicate expression levels in HPTC (in some cases expression levels were very low and the bar was not discernible). The markers examined are indicated, as well as their *in vivo* expression patterns (pluripotent stem cells (PSC), mesoderm, mesenchymal renal precursors, nephron progenitors, PTC). All gene IDs, descriptions and acronyms of markers examined by qPCR are summarized in the Supplementary Table S1. (**b**) The expression levels of 31 markers were determined by qPCR in undifferentiated iPS(Foreskin)-4 cells (white bars), iPS(Foreskin)-4-derived HPTC-like d8 cells (gray bars) and in HPTC (black bars). The bars show the means +/− s.d. (n = 3). Markers were grouped according to their functions or *in vivo* expression patterns as indicated at the bottom. (**c**–**h**) Functional morphology of iPS(Foreskin)-4-derived d8 cells. (**c**–**e**) Domes formed by cells cultivated on tissue culture plastic (TCPS). Different focal planes are displayed in panels (**c**–**e**) GLUT1 (green) was detected by immunofluorescence. Scale bars: 100 μm. (**f**) Cells were polarized with an apical brush border (scale bar: 5 μm). Tubules generated by cells cultivated on the surface of Matrigel (**g**) or TCPS (**h**). Scale bars: 100 μm.

iPS(Foreskin)-4 cells were differentiated by cultivating the cells in matrigel-coated multi-well plates with renal epithelial cell growth medium (REGM) supplemented with bone morphogenetic protein (BMP)2 and BMP7 (Supplementary Fig. S1, for details see Methods). Changes in gene expression patterns were monitored (Fig. 1a). OCT3/4, NANOG, SOX2 and DNMT3B were down-regulated after day (d) 1 (all gene IDs, descriptions and acronyms of markers examined by qPCR are summarized in the Supplementary Table S1). Down-regulation of these “stemness” markers was followed by a transient pulse of T on d3 (Fig. 1a). T is transiently expressed in the early mesoderm of vertebrate embryos^14^, from which the kidneys are derived.

OSR1 became strongly up-regulated between d7 and d9 (Fig. 1a). During embryonic development OSR1 is continuously expressed throughout development in the renal precursor population^15^. Between d7 and d9 nephron progenitor and PTC markers also became strongly up-regulated (Fig. 1a). These included the nephron progenitor markers SIX2^16^, WT1^17^ and GDNF^18^, as well as HOXD11, which specifies metanephric kidney development^19^. PTC markers that were up-regulated between d7 and d9 were KSP-CAD, AQP1 and GGT. The expression of KSP-CAD is kidney-specific, and limited to tubular epithelial cells^20^. In the kidney AQP1 and GGT expression is characteristic for proximal tubular epithelial cells^21^,^22^.

These results showed a profound change between d7 and d9, and on d9 the cells expressed high levels of all of the tested nephron progenitor and PTC markers. Although nephron progenitor markers are not expressed in mature human and murine PTC *in vivo*, they become typically re-expressed under *in vitro* conditions^2^,^23^,^24^. This was confirmed by the results obtained here showing that *in vitro* cultures of HPTC (Fig. 1a; black bars) expressed a similar pattern of nephron progenitor and PTC markers as hiPSC-derived cells on d9.

The observed changes in gene expression, which included early down-regulation of stemness markers, a transient peak of T and subsequent up-regulation of OSR1 and nephron progenitor markers, were in agreement with other recent results on the differentiation of pluripotent stem cells towards the renal lineage^8^,^9^,^11^,^12^,^13^. In most of these studies up-regulation of markers expressed in terminally differentiated renal cells was also observed, as in our case.

Overall, the results showed that after day 7 PTC markers were co-expressed with nephron progenitor markers, and also stemness markers remained to be expressed to a certain degree (Fig. 1a). Expression of NANOG and SOX2 was also detected in HPTC (Fig. 1a). In this respect it is interesting to note that expression of the pluripotent stem cell marker OCT3/4 was observed in some tubular cells in the adult kidney^25^, and expression of multi-lineage and nephron progenitor markers was seen in PTC in the adult kidney after injury after these cells had achieved full differentiation^24^,^26^. Thus, the expression of stemness, multi-lineage and nephron progenitor markers is not restricted to embryonic development, and the flexibility of PTC with respect to the expression of such markers seems to be related to proliferation and their roles in tissue regeneration^24^,^26^.

For the successful development of *in vitro* applications it is important to take into account that PTC can react flexibly to the environmental and proliferation conditions and may display features that are usually not observed under normal *in vivo* conditions. A broader characterization of cellular features allows an informed decision of whether potential changes would interfere with certain applications, and to what extent the cells display typical features of PTC. Therefore, we characterized in the following morphological features and tubulogenesis, gene and protein expression patterns, enzymatic activities, drug transporter activity, drug transporter-dependent induction of gene expression, cellular responses to nephrotoxic and not nephrotoxic compounds and drug-induced cellular signalling pathways and damage responses.

### Characterization of hiPSC-derived cells

Marker expression patterns of iPS(Foreskin)-4-derived d8 cells were characterized in detail by qPCR. To determine the expression levels of 31 markers, we used another independently differentiated batch of such hiPSC-derived HPTC-like cells. Undifferentiated iPS(Foreskin)-4 cells and HPTC were used for comparison (Fig. 1b). The results confirmed that KSP-CAD was expressed in HPTC-like cells and HPTC at at least ~100-fold higher levels than in undifferentiated hiPSC. The same applied to SLC34A1. This gene codes for a type II sodium/phosphate co-transporter, which is expressed only in fully differentiated PTC^27^. Also other PTC-specific transporters which are involved in sodium, bicarbonate and glucose transport (NBC1, SGLT2 and GLUT5) were expressed in HPTC and HPTC-like cells. In addition, all drug transporters tested were expressed in HPTC and HPTC-like cells. The expression levels of the main organic anion uptake transporters, OAT1 and OAT3^28^, were ~15-fold (P = 0.0004) or ~5-fold (P = 0.0157) higher in hiPSC-derived HPTC-like cells, respectively (compared to HPTC). About 2-fold higher expression levels were observed in case of OCT2 (P = 0.0005), which is important for uptake of various nephrotoxicants by PTC, including cisplatin^29^. Further tests confirmed that OCT2 was functional (Supplementary Fig. S2). PEPT1 and MEG were expressed at ~2-fold (P = 0.0001) and ~26-fold (P = 0.0041) higher levels HPTC-like cells (compared to HPTC; Fig. 1b). MEG is important for the uptake of nephrotoxic aminoglycoside antibiotics. The organic cation uptake transporter OCTN2 was expressed at similar levels in HPTC and HPTC-like cells (P = 0.1608) and the efflux transporter MDR1 was expressed at higher levels in HPTC (~39-fold; P = 0.0003).

Also a variety of other PTC-specific markers (AQP1, CD13, GGT, VIT D3, Na^+^K^+^ ATPase) were expressed in iPS(Foreskin)-4-derived HPTC-like cells and in HPTC, as well as epithelial markers (ZO-1, N-CAD, E-CAD; Fig. 1b). GGT expression was confirmed by functional assays (Supplementary Fig. S2). PAX2 and WT1 were expressed as well in HPTC and HPTC-like cells (Fig. 1b). These two markers are expressed in renal and nephron progenitors and in HPTC and primary animal PTC cultivated *in vitro*^5^,^23^,^24^,^30^. Expression of PAX2 and WT1 was confirmed at the protein level (Supplementary Fig. S3). In addition, expression of kidney injury markers (NGAL^31^,^32^, KIM-1^32^,^33^, VIM^34^) and SMA was observed (Fig. 1b). Various studies have shown that expression of kidney injury markers, as well as of E-CAD and SMA, is typical for HPTC and primary animal PTC under *in vitro* conditions^2^,^5^,^23^,^24^,^34^,^35^,^36^. PAX2 and NGAL were expressed at about 34-(P = 0.00003) or 122-fold (P = 0.0002) lower levels in HPTC-like cells in comparison to HPTC, and NGAL expression was not upregulated during hiPSC differentiation. Also KIM-1 and VIM were expressed at about 3-(P = 0.0002) to 4-fold (P = 2.4 × 10^−7^) lower levels in HPTC-like cells (compared to HPTC).

Another typical feature of *in vitro* cultivated HPTC, as well as of hESC-derived HPTC-like cells, is the expression of markers that are expressed in other renal cell types *in vivo*^6^. Here, we observed expression of PODXL, NCCT, NKCC2, UMOD and AQP3 in both, HPTC and hiPSC-derived HPTC-like cells (Fig. 1b). PODXL is expressed in renal podocytes as well as in undifferentiated hiPSC derived from normal human fibroblasts^37^. Here, PODXL expression was highest in undifferentiated hiPSCs, and ~100-fold higher in HPTC-like cells than in HPTC (Fig. 1b). This marker was probably not substantially down-regulated during hiPSC differentiation, and hence the relatively high expression levels in HPTC-like cells are unlikely to reflect differentiation into podocyte-like cells. This is consistent with the finding that also no other characterisitic features of podocytes were observed during further characterization as, for instance, with respect to cell morphology (Fig. 1c–h).

**Figure 2:**
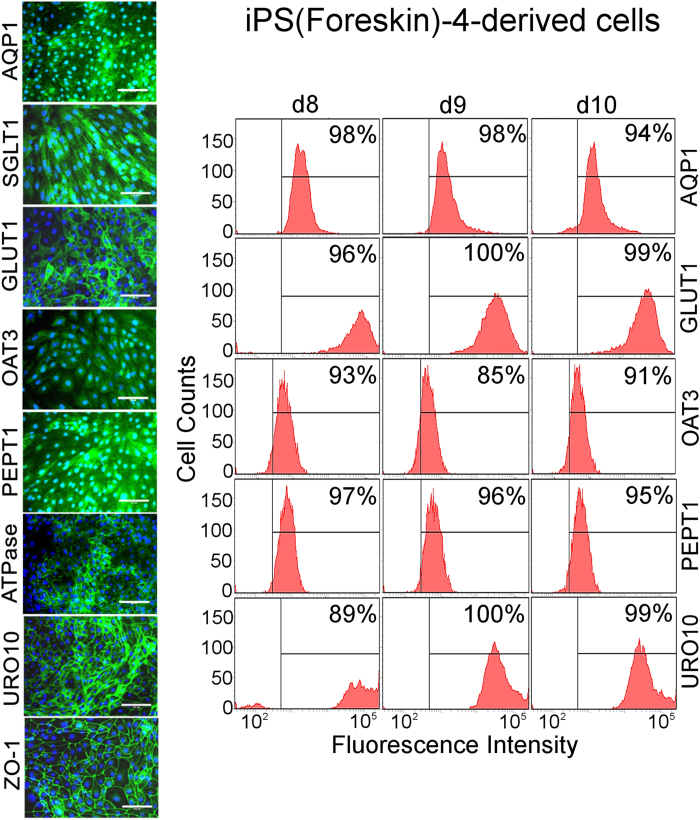
Characterization of hiPSC-derived cells by immunostaining and FACS. The left-hand panels show epithelia of d8 cells derived from iPS(Foreskin)-4 cells. The PTC-specific markers indicated on the left were detected by immunofluorescence (green: markers, blue: nuclei): Scale bars: 100 μm. The right-hand panels show FACS results obtained with iPS(Foreskin)-4-derived cells harvested on day 8, day 9 and day 10. The percentages of cells positive for the markers indicated on the right are displayed. The FACS results were consistent with the immunostaining data and showed that in most cases >90% of the cells were positive.

We confirmed by immunostaining of iPS(Foreskin)-4-derived d8 cells that the PTC marker AQP1 and the collecting duct marker AQP3 were co-expressed by the same cells (Supplementary Fig. S4). Therefore, mixed marker expression patterns were not due to the presence of different cell populations. This was consistent with the fact that in most cases >90% of cells expressed PTC-specific markers (Fig. 2 and Supplementary Fig. S5). AQP1 and AQP3 are water channels that are normally enriched in the cell membrane. The immunostaining results revealed surface enrichment of the PTC marker AQP1, whereas the collecting duct marker AQP3 co-expressed by the same cells showed aberrant sub-cellular localization and no enrichment at the cell surface (Supplementary Fig. S4).

Expression of 13 markers, including 10 PTC-specific markers, was also determined in iPS IMR90-4- and iPS DF19-9-11T.H-derived d8 cells by qPCR (Supplementary Fig. S6). Most of the markers were also expressed in cells derived from these two hiPSC lines. However, in most cases marker expression levels were highest in iPS(Foreskin)-4-derived cells (Supplementary Fig. S6). Activity of the brush border enzyme GGT was confirmed at the functional level in iPS IMR90-4- and iPS DF19-9-11T.H-derived cells (Supplementary Fig. S7). Expression of the brush border enzyme CD13 was confirmed at the protein level by immunostaining in iPS IMR90-4-derived d8 cells (Supplementary Fig. S7).

Next, we characterized morphological features of iPS (Foreskin)-4-derived d8 cells. In addition to displaying gene expression patterns (Fig. 1) and functional features (GGT and OCT2 activity; Supplementary Fig. S2) that are characteristic for HPTC, iPS(Foreskin)-4-derived d8 cells also displayed a variety of typical morphological features. These included dome formation, polarization with an apical brush border and tubulogenesis (Fig. 1c–h; similar processes of tubulogenesis on Matrigel and tissue culture plastic have been observed with HPTC^36^ and hESC-derived HPTC-like cells^6^).

Immunostaining experiments repeated with independently differentiated batches of iPS(Foreskin)-4-derived d8 cells revealed formation of confluent renal epithelia with tight junctions (chicken wire-like ZO-1 patterns; Fig. 2). The images suggested that almost all cells expressed the PTC-specific marker proteins AQP1, SGLT1, GLUT1, OAT3, PEPT1, Na^+^/K^+^ ATPase, URO10 and ZO-1 (Fig. 2). Quantitative image analysis that was performed with respect to six of these markers confirmed expression in at least ~90% of the cells (Supplementary Fig. S5). In all cases enrichment of the marker proteins at the cell surfaces was observed (Fig. 2 and Supplementary Fig. S8). Some markers showed additional staining distributed over the cell area. This would be expected for epifluorescence images because, unlike ZO-1, most of the marker proteins do not localize only at the lateral cell surfaces, but normally localize mainly at the apical brush border (SGLT1, PEPT1), baso-lateral membranes (OAT3) or apical and baso-lateral membranes (AQP1).

Whereas there was no indication that there were major problems with aberrant subcellular localization of marker proteins in iPS(Foreskin)-4- and iPS DF19-9-11T.H-derived cells (Fig. 2 and Supplementary Figs. S8 and S9), the staining patterns of some markers were not always in agreement with the expected subcellular localization in case of iPS IMR90-4-derived d8 cells (Supplementary Fig. S10). For instance, SGLT1 was mainly localized in the nuclear and peri-nuclear areas. However, also in case of iPS IMR90-4-derived d8 cells various markers like GLUT1, Na^+^/K^+^ ATPase, URO10 and ZO-1 showed the expected sub-cellular localization with cell surface enrichment. Also immunostaining results obtained with IMR 90-4-derived cells were analyzed by quantitative image analysis in order to determine the numbers of positive cells (regardless of subcellular localization, Supplementary Fig. S11). Five markers were analyzed and in all cases protein expression was observed in >90% of the cells.

In addition, marker expression was also analyzed by FACS in iPS(Foreskin)-4- and iPS IMR90-4-derived cells (Fig. 2 and Supplementary Fig. S10). Five different markers were anlayzed in three independent experiments after cells were harvested on day 8, day 9 or day 10 of differentiation. The results showed consistently expression in high percentages of cells, which were in most cases ~90% and above. These results also showed that PTC markers were expressed in most of the cells at least until day 10. Similar results were obtained with d8 and d9 cells derived from iPS DF19-9-11T.H cells (Supplementary Fig. S9). In this case expression of six different markers was determined using FACS.

Due to the high purity of hiPSC-derived d8 cells these cells could be directly used for subsequent *in vitro* applications without the need for harvesting or further purification. As overall PTC marker expression levels were highest in iPS(Foreskin)-4-derived cells (Supplementary Fig. S6) and subcellular protein localization was as expected (Fig. 2 and Supplementary Fig. S8), iPS(Foreskin)-4-derived cells were selected for further analyses.

### Transporter-mediated drug uptake and drug-induced interleukin expression

**Figure 3:**
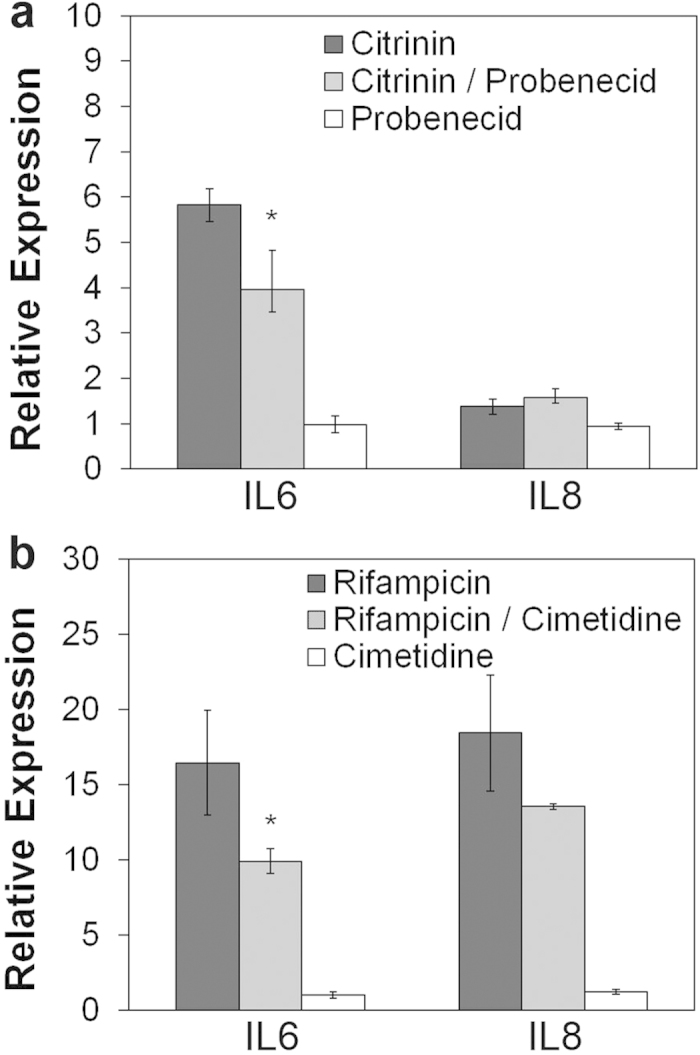
Transporter-mediated drug uptake and drug-induced expression of IL6 and IL8. iPS(Foreskin)-4-derived d8 HPTC-like cells were treated with 100 μg/ml of (**a**) citrinin or (**b**) rifampicin. IL6 and IL8 expression levels in drug-treated cells are indicated by the dark-gray bars. The bars show the mean +/− s.d. (n = 3) and all expression levels were normalized to the vehicle controls, which were set to 1. Citrinin uptake by PTC is mediated by OAT1 and OAT3 and these transporters are inhibited by probenecid. Exposure to citrinin and probenecid (light-grey bars in panel a) reduced the levels of citrinin-induced IL6 expression by 31%. Rifampicin uptake by PTC is mediated by OCT2, which is inhibited by cimetidine. Exposure to rifampicin and cimetidine reduced the levels of rifampicin-induced IL6 and IL8 expression by 40% and 26%, respectively (light-gray bars in panel b). The inhibitors alone did not significantly alter IL6 and IL8 expression levels relative to vehicle controls (white bars; both inhibitors were used at a concentration of 2 mM). Significant differences between drug-treated and drug + inhibitor-treated samples are indicated by asterisks.

Compounds that are toxic for PTC specifically increase IL6 and/or IL8 expression in HPTC and hESC-derived HPTC-like cells^5^,^7^. Based on this characteristic, a method was developed for the prediction of PTC toxicity in humans^5^,^7^. Here, we tested whether compounds that are toxic for PTC also increase IL6 and/or IL8 expression in iPS(Foreskin)-4-derived d8 cells. The cells were differentiated as usual in multi-well plates, and were treated on the evening of day 8 for 16 hours with the PTC-specific nephrotoxicants citrinin and rifampicin. IL6 and IL8 levels were determined subsequently by qPCR (a flow chart of the procedures is provided in the Supplementary Fig. S1). Rifampicin increased IL6 expression ~17-fold and IL8 expression ~18-fold (Fig. 3). Citrinin increased IL6 expression ~6-fold, whereas no increase in IL8 expression was observed (Fig. 3). Previous results revealed that citrinin also did not induce IL8 in hESC-derived HPTC-like cells, and typically not every drug induces both interleukins^5^,^7^.

Citrinin uptake by PTC is mediated by OAT1 and OAT3^38^. These transporters are inhibited by probenecid^38^. Co-incubation with citrinin and probenecid reduced the level of citrinin-induced IL6 expression significantly by ~31% (Fig. 3). This result revealed that citrinin uptake was mediated by OAT1 and OAT3, in agreement with the expression of these transporters in iPS(Foreskin)-4-derived d8 cells (Figs 1 and 2).

Similar experiments were performed with rifampicin. Uptake of this drug by PTC is mediated by OCT2, which is inhibited by cimetidine^39^. Co-incubation with cimetidine reduced the rifampicin-induced increase of IL6 and IL8 expression by 40% and 26%, respectively (Fig. 3). These results suggested that rifampicin-induced induction of IL6 and IL8 was dependent on transporter-mediated uptake of the drug. The results were in agreement with expression (Fig. 1) and activity (Supplementary Fig. S2) of OCT2 in iPS(Foreskin)-4-derived d8 cells.

### Predictive performance

Next, we addressed whether PTC-specific nephrotoxicity of drugs could be predicted with hiPSC-derived HPTC-like cells. This question was addressed with the IL6/IL8-based assay^5^,^7^ (for overall procedure see Supplementary Fig. S1). Briefly, iPS(Foreskin)-4-derived d8 cells were exposed overnight to 30 compounds (Supplementary Table S2). These could be divided into two groups. Group 1 contained 18 nephrotoxicants that are directly toxic for PTC in humans (Supplementary Table S2). Group 2 contained compounds that are not toxic for PTC in humans. This group comprised 4 non-nephrotoxic compounds and 8 nephrotoxicants that do not directly damage PTC (Supplementary Table S2). Detailed information on the nephrotoxicity of the compounds in humans has been provided^7^. After overnight exposure, changes in the levels of IL6 and IL8 were determined by qPCR. Supplementary Tables S2 and S3 show the results on IL6 and IL8 expression levels for all the 30 compounds at all tested concentrations. All compounds were blinded during testing.

**Figure 4:**
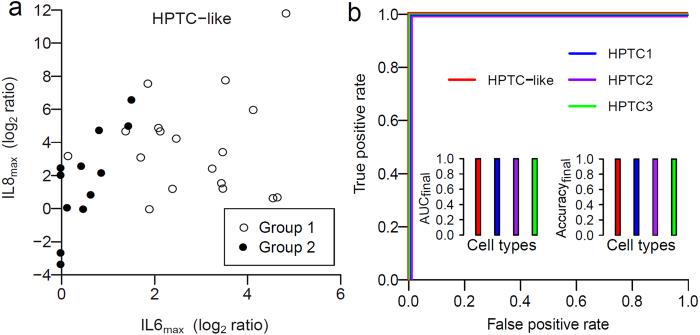
Automated classifiers based on the random forest algorithm could perfectly classify compounds from group 1 and 2 that are not linearly separable. (**a**) Scatter plot showing changes in the IL6 and IL8 gene expression levels of hiPSC-derived HTPC-like cells induced by compounds from group 1 (white; toxic for PTC in humans) and group 2 (black; not toxic for PTC in humans). Each dot represents a compound. The shown values were used to train a random forest (RF) classifier. (**b**) The receiver operating characteristic (ROC) curves of the final RFs trained on all data collected from hiPSC-derived HPTC-like cells (red), and three batches of HPTC (HPTC1 - blue, HPTC2 - purple, and HPTC3 - green). The four graphs overlap. The insets provide the values for the area under the curve (AUC_final_) and balanced accuracy (Accuracy_final_) of the classifiers. RFs based on the HPTC-like cells and HPTC can perfectly separate the compounds.

We compared the performance of hiPSC-derived HPTC-like cells to HPTC, which have been tested previously by us^5^,^40^. In order to have a fair comparison, we needed to use similar numbers of compounds. Therefore, we re-computed the performance of HPTC using 29 out of the original 41 compounds tested previously. For each compound in both HPTC-like cells and HPTC, we used log-logistic models to estimate its IL6 and IL8 dose response curves, and determined the responses at the highest tested doses from the estimated curves (IL6_max_ and IL8_max_, Fig. 4a). Based on these features, an automated classifier called random forest (RF)^40^ was used to classify the compounds as toxic or not toxic for PTC (in comparison to other classifiers, RF showed the best performance when tested with HPTC^40^). Finally, we used a cross validation procedure to randomly divide all the compounds into two non-overlapping subsets, train a classifier on one of the subsets, and tested the trained classifier on the other unused subset. The training and test accuracies were measured from the training and test subsets, respectively.

We found that hiPSC-derived HPTC-like cells have similar training accuracy (~99.9%) but a higher test accuracy (87.0% vs 82.0%) than the HPTC (Table 1). That means our models can almost perfectly separate the toxic and non-toxic compounds in the training data, and also a high test accuracy can be achieved with HPTC-like cells. We also trained a final RF classifier using all the compounds, and found that the classifier, as expected, can perfectly separate the two groups (final accuracy of 100%, Fig. 4b) for both HPTC-like cells and HPTC. Importantly, we also found that HPTC from three different donors (HPTC1, 2, and 3) gave highly variable prediction performances. For instance, the specificity may range from ~64.5% to ~91.5% (Table 1). The use of hiPSC-derived HPTC-like cells helps to avoid problems with inter-donor variability, as well as other issues associated with the use of primary cells, such as cell sourcing problems and functional changes during passaging. Together, our results showed that hiPSC-derived HPTC-like cells could be used to predict PTC-specific nephrotoxicity of drugs.

### Drug-induced injury mechanisms

Next, we addressed whether not only drug-induced toxicity could be predicted, but also underlying injury mechanisms and compound-induced cellular pathways. The compounds used in these experiments were acarbose, ethylene glycol, aristolochic acid and cisplatin. All compounds were tested at the same concentrations as used in the IL6/IL8-based assay.

We addressed compound-induced generation of DNA double strand breaks, reactive oxygen species (ROS) generation and inflammation by using γH2AX generation, 4-hydroxynonenal production (4-HNE; ROS-induced lipid peroxidation product) and nuclear-cytoplasmic translocation of the nuclear factor (NF)-κB p65 subunit as endpoints. All biomarkers were detected by immunofluorescence, which is compatible with automated cellular imaging. Of note, the cells were fixed and stained in the morning of d9 in the same multi-well plates used for cell differentiation, and the overall procedure involving cell differentiation, compound treatment, biomarker detection and automated imaging could be completed within 9 days (Supplementary Fig. 1).

**Figure 5:**
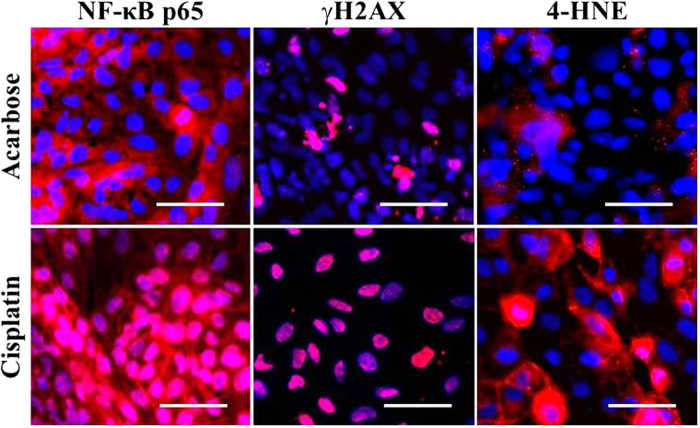
Drug-induced injury mechanisms and cellular pathways detected with automated cellular imaging. hiPSC-derived HPTC-like cells were treated with increasing concentrations of acarbose and cisplatin. Results obtained with the maximal concentrations (1000 μg/ml) are shown. NF-kB p65, γH2AX and 4-HNE were detected by immunostaining (red). Cell nuclei were stained with DAPI (blue). Representative images captured with a high content analyzer (NF-κB p65 and γH2AX) or an automated microscope (4-HNE) are shown. Nuclear enrichment of NF-κB p65 and γH2AX and increased 4-HNE content in the cell membrane were induced by cisplatin. Scale bars: 50 μm.

All results remained negative with respect to acarbose and ethylene glycol up to the highest concentration tested (1000 μg/ml, Fig. 5 and Table 2). This was consistent with the fact that these compounds are not toxic for PTC in humans. Acarbose is an α-glucosidase inhibitor used for the treatment of type 2 diabetes mellitus. In humans adverse effects on different organ systems including liver, lung and skin have been described (http://chem.sis.nlm.nih.gov/chemidplus/rn/56180-94-0#toxicity), but to our knowledge no toxic effects on the renal proximal tubule have been observed.

Ethylene glycol has toxic effects on various human organ systems, in particular on the peripheral and central nervous system and sense organs (http://chem.sis.nlm.nih.gov/chemidplus/rn/107-21-1#toxicity). In addition, the gastrointestinal tract, lungs, liver, bladder, urether and kidney can be affected. Ethylene glycol damages the kidney through the formation of calcium oxalate monohydrate crystals in the proximal tubules (~90% of the water from the glomerular filtrate is reabsorbed in the proximal tubules). Such crystals were observed in the proximal tubules of rats and human patients, and crystal formation rather than direct toxicity of the compound leads to PTC damage^41^,^42^. Of note, crystal formation does not occur *in vitro* where the water concentration is constant. The results obtained here with ethylene glycol show the high specificity of the hiPSC-based *in vitro* model with respect to detecting direct toxic effects on PTC, and confirm that other kinds of adverse effects that may occur *in vivo* cannot be detected in these ways.

The results obtained here with aristolochic acid and cisplatin revealed generation of DNA double strand breaks and ROS as detected by significantly increased nuclear γH2AX and 4-HNE levels (Fig. 5 and Table 2). This occurred in conjunction with induction of an inflammatory response (nuclear translocation of NF-κB p65; Fig. 5 and Table 2). The latter result was consistent with a marked increase in the expression of the pro-inflammatory cytokines IL6 and IL8 in response to these compounds (~3- to 44-fold; Supplementary Tables S2 and S3. These results were in concordance with clinical data, which have shown that aristolochic acid (a compound used in traditional Chinese medicine) induces AKI or chronic tubulointerstitial nephropathy and urothelial cancers in human patients^43^,^44^. Direct toxic effects on the proximal tubules of humans and experimental animals are associated with necrosis of tubular cells and a profound inflammatory response originating in these areas^45^. The carcinogenicity of aristolochic acid is due to its DNA-damaging properties resulting in the formation of DNA adducts and DNA double strand breaks^46^. Part of the aristolochic acid-induced DNA damage is due to oxidative stress and ROS generation^47^.

Also the results obtained here with cisplatin were in agreement with clinical data and the results of animal experiments. The anti-cancer drug cisplatin has dose-limiting nephrotoxicity and is directly toxic for the proximal tubules of humans and experimental animals. Cisplatin-induced PTC injury is due to damage of nuclear and mitochondrial DNA after transporter-mediated uptake^29^,^48^. This is associated with a profound inflammatory response of the proximal tubule, release of pro-inflammatory cytokines and interleukins and ROS generation^29^,^49^,^50^. Together, the results showed that various compound-induced pathways and injury mechanisms associated with direct PTC toxicity in humans were specifically activated and correctly detected with the hiPSC-based renal *in vitro* model.

In summary, we have established a rapid and simple 1-step protocol for the differentiation of hiPSC into HPTC-like cells and the first hiPSC-based renal *in vitro* model suitable for compound screening. Due to the high purity (>90%) of the hiPSC-derived HPTC-like cells the same multi-well plates could be efficiently used for cell differentiation and subsequent drug testing on day 8, without the need of harvesting or purifying the cells in between. All cell differentiation and compound testing procedures could be completed within 9 days. We combined for the first time a stem cell-based renal *in vitro* model with machine learning methods for nephrotoxicity prediction. Automated and unbiased data analysis in combination with the use of hiPSC-derived HPTC-like cells resulted in 99.8% training balanced accuracy and 87.0% test balanced accuracy with respect to predicting proximal tubular toxicity in humans. Further, drug-induced cellular pathways and injury mechanisms that are known to be associated with proximal tubular toxicity in humans could be specifically activated and correctly identified with hiPSC-derived HPTC-like cells.

The technology developed here will also enable the development of personalized or disease-specific *in vitro* cell-based models for nephrotoxicity screening and prediction. For such approaches screening technologies based on hiPSC-derived cells are essential. Cell samples from patients affected by kidney disease or adverse drug effects could be obtained from skin, blood or urine^51^ and differentiated into HPTC-like cells after reprogramming into hiPSC. Screening of such cells would allow personalized toxicity prediction and would facilitate the identification of genetic variants associated with adverse drug effects. Further, patient-specific hiPSC-derived renal cells would facilitate the development of personalized therapies.

## Methods

### Expansion and differentiation of hiPSC

iPS(Foreskin)-4, iPS IMR90-4 and iPS DF19-9-11T.H cells were obtained from the WiCell Research Institute (Madison, WI, USA). The work with these hiPSC lines was approved by the Institutional Review Board of the National University of Singapore (NUS-IRB reference code: 13–437). Undifferentiated cells were expanded with mTeSR1 medium (Stemcell Technologies, Singapore) in multi-well plates coated with growth factor-reduced Matrigel (BD, Franklin Lakes, NJ, USA). For differentiation, hiPSC were seeded (day 0) at a density of 8000 cells/cm^2^ into 24-well plates coated with growth factor-reduced Matrigel. Single cell suspensions were prepared with StemPro Accutase (Merck Millipore, Billerica, MA, USA). Cells were resuspended and seeded with commercial renal epithelial growth medium (REGM BulletKit; Lonza Bioscience, Singapore) supplemented with 10 μM Rho kinase (ROCK) inhibitor (Y-27632, Calbiochem, Merck, Darmstadt, Germany). The medium was exchanged on day 1 against REGM supplemented with 10 ng/ml of bone morphogenetic protein (BMP)2 (Sigma-Aldrich, St. Louis, MO, USA) and 2.5 ng/ml of BMP7 (Life Technologies, Carlsbad, CA, USA). The BMP-supplemented medium did not contain ROCK inhibitor. The medium was exchanged every other day. Compound treatment was performed on day 8 and the same plates in which the cells had been differentiated were continued to be used for compound testing. A flow chart of the differentiation procedure with subsequent drug testing is provided in the Supplementary Fig. S1. Various aspects of the differentiation protocol used here were different in comparison to a previously published protocol^6^ (for details of the previously published protocol see^6^). The seeding density and the use of ROCK inhibitor in the current protocol were important for improved cell survival and differentiation rates.

### HPTC

One lot of HPTC was obtained from the American Type Culture Collection (ATCC, Manassas, VA, USA). This lot is called HPTC1 in Table 1 and was also used for all of the other experiments shown in the other display items. This lot was cultivated and used at passages 4 and 5 as before^5^,^6^,^7^. Two additional lots of HPTC (HPTC2 and 3 in Table 1) were obtained from nephrectomy samples from tumor patients. Areas with normal non-tumor tissue were identified by a pathologist and anonymized normal tissue samples were obtained from the Tissue Repository of the National University Health System (NUHS, Singapore). HPTC were isolated from these tissue samples and used at passages 3 and 4 as described before^5^. The Institutional Review Board’s approval for the work with human kidney samples (DSRB-E/11/143) and commercial HPTC (NUS-IRB Ref. Code 09-148E) has been obtained.

### Fluorescence-activated cell sorting (FACS)

FACS was performed as outlined before^6^. Primary antibodies against AQP1 and GLUT1 were purchased from Abcam (Cambridge, MA, USA) and primary antibodies against PEPT1, OAT3 and URO10 were obtained from Santa Cruz Biotechnology Inc. (Santa Cruz, CA, USA). Secondary anti-mouse or anti-rabbit antibodies conjugated to Alexa Fluor 488 were used (Life Technologies, Carlsbad, CA, USA). Control cells were incubated with the secondary antibody only. 50,000 cells were analyzed per sample.

### Immunofluorescence

Immunofluorescent staining was performed as described^5^ and primary antibodies against AQP1, SGLT2, GLUT1, SMA and PAX2 were purchased from Abcam. Primary antibodies against OAT3, CD13, URO10, AQP3, SGLT1, PEPT1, Na^+^/K^+^ ATPase and WT1 were obtained from Santa Cruz Biotechnology Inc. and an antibody against ZO-1 was purchased from Invitrogen (Carlsbad, CA, USA). Secondary CY3- or Alexa Fluor 488-labeled goat anti-rabbit or anti-mouse antibodies were obtained from Life Technologies.

### Quantitative analysis of immunofluorescence images

After immunostaining and imaging by epifluorescence microscopy the numbers of positive cells that express a marker were determined by using quantitative image analysis. For background correction the ImageJ software (NIH) was used (Rasband, W.S., ImageJ, US National Institutes of Health, Bethesda, Maryland, USA, http://imagej.nih.gov/ij/, 1997–2014). Segmentation and measurement of the mean intensity (*μ_i_*) value for each cell was performed by using the cellXpress software platform (v1.2, Bioinformatics Institute)^52^. All intensity values were log10-transformed. The threshold for distinguishing the positive and negative cells was determined semi-automatically. First, a background region was manually selected from the images, and its mean intensity (*M_b_*) and standard deviation (*Σ_b_*) values were measured. Second, we assumed that the background intensity values are approximately normally distributed. The 90 percentile of the normal distribution was used as a threshold for selecting positive cells. This is equivalent to setting a threshold according to equation 1:


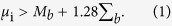


### Determination of marker expression levels

Marker expression levels were determined by qPCR as described^5^. Details of primers and amplicons are provided in the Supplementary Table S1.

### Transporter-mediated drug uptake and interleukin induction

In the experiments related to Fig. 3, drug treatment was performed overnight for 16 hours as usual. Inhibitors were added 30 minutes before the drugs.

### Scanning electron microscopy (SEM)

SEM was performed as outlined before^6^.

### GGT activity

γ-glutamyltransferase 1 (GGT) activity was determined as described^6^. All data were normalized to the protein content of the cells. The Pierce bicinchoninic acid (BCA) protein assay kit (Thermo Scientific, Rockford, IL, USA) was used to quantify the amount of protein in cell extracts.

### Drug transporter activity

OCT2 activity was measured as outlined before^2^ by using 25 μM of ASP^+^ (4-(4-(dimethylamino)styryl)-N-methylpyridinium iodide). Data were analyzed by calculating multidrug resistance”: ...calculating multidrug resistance activity factor (MAF) activity factor (MAF) values^53^. Samples with MAF values >25 were considered as being positive for transporter activity.

### Compound treatment and determination of IL6 and IL8 expression levels

Compound treatment was performed for 16 hours. All compounds, except aristolochic acid, have been used in our previous studies^5^,^7^ and detailed information on their nephrotoxicity in humans has been provided^7^. Aristolochic acid (a 1:1 mixture of aristolochic acids I and II) was purchased from EMD Millipore (Billerica, MA, USA). All compounds were tested at concentrations of 1, 10, 100 and 1000 μg/ml (three replicates each). All results were normalized to the vehicle controls as described^5^,^7^. All plates contained as controls (three replicates each) 100 μg/ml dexamethasone (negative) and 100 μg/ml puromycin (positive). Z’ values were calculated as described^54^ and plates with Z’ values >0.5 were included. IL6 and IL8 mRNA levels were determined by quantitative real-time reverse transcription polymerase chain reaction (qPCR) as before^5^,^7^ by using the same primers (Supplementary Table S1). All data were normalized to two reference genes (GAPDH and PPIA).

### Automated cellular imaging

hiPSC were differentiated in 96-well plates and cells were fixed in the morning of day 9 after compound treatment for 16 hours. NF-κB p65, γH2AX and 4-HNE were detected with primary antibodies from Abcam. Alexa Fluor 488-conjugated goat anti-rabbit or anti-mouse antibodies were used as the secondary antibodies (Life Technologies). Cell nuclei were stained with 4′,6-diamidino-2-phenylindole (DAPI). In case of NF-κB p65 and γH2AX the cells were imaged with the ImageXpress^MICRO^ system (Molecular Devices, UK) using the MetaXpress Image Acquisition and Analysis Software version 2.0. Nine sites per well were imaged. The images were analysed automatically by the MetaXpress software and for determining nuclear translocation of NF-kB p65 the Translocation-Enhanced Module^55^ was used. Automated imaging of cells stained for 4-HNE was performed with a Zeiss AxioObserver Z1 microscope (Carl Zeiss AG, Jena, Germany) using Zeiss AxioVision Rel. 4.8.2 software. Nine images were acquired per well and channel and image analysis was performed with the CellXpress 1.2 software^52^. Based on results obtained with positive (100 μg/ml puromycin or gold chloride) and negative (100 μg/ml dexamethasone) controls the Z’ values were calculated^54^ and plates with values >0.5 were included.

### Calculations and Statistics

All calculations and statistics that were not part of the computational analysis described below were performed with Microsoft Office Excel 2010. The base-2 logarithm (log_2_) of the average fluorescence intensities was calculated with respect to γH2AX and 4-HNE. The one-sample t-test was used to determine significant differences. The normal distribution of the data was confirmed using SigmaStat (3.5) (Systat Software Inc., Chicago, IL, USA).

### Computational analysis

For each drug, we normalized the IL6 or IL8 expression values measured at all doses to the respective vehicle controls. Then, we applied log2 transformation to the resulting ratios, and used a three-parameter log-logistic model with lower limit = 0 to obtain a sigmoidal dose response curve^56^. The model is described by equation 2:


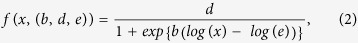


where *x* is the drug concentration, *e* is the response half-way between the upper limit *d* and 0, and *b* is the relative slope around *e*. From the estimated dose response curve, we determined the response value (IL6 or IL8 levels) at the highest tested drug dosage (IL6_max_ or IL8_max_). Data on the expression of IL6 and IL8 in three batches of HPTC had been obtained previously^5^ and were re-analyzed here (HPTC were tested here on 29 out of the 30 compounds).

We used random forest (RF)^40^ to predict drug-induced nephrotoxicity. The RF has two parameters: number of decision trees (B) and number of features (*m*_*rf*_). We optimized these parameters using an exhaustive grid search for B = 10, 50, 150, 250, 400, 500, and *m*_*rf*_= 1, 2, 3, 4, 5.

Finally, we used a 10-fold cross validation procedure to estimate classification performance^57^. We randomly divided the whole datasets into 10 roughly equal and stratified folds, 9 of which were used to train the RF and the remaining fold to test the trained RF. The whole procedure was repeated 10 times. All the classification performance measurements were averaged from these 10 trials. We used the following three classification performance measurements (equations 3–5):


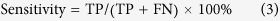



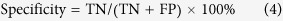



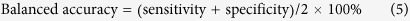


TP is the number of true positives, TN is the number of true negatives, FP is the number of false positives and FN is the number of false negatives. All the analyses were performed using the ‘randomForest’ library (v4.6-10) under the R statistical environment (v3.0.2) on a personal computer equipped with an Intel Core i7-3770K processor and Windows 7 operating system.

## Additional Information

**How to cite this article**: Kandasamy, K. *et al.* Prediction of drug-induced nephrotoxicity and injury mechanisms with human induced pluripotent stem cell-derived cells and machine learning methods. *Sci. Rep.* **5**, 12337; doi: 10.1038/srep12337 (2015).

## Supplementary Material

Supplementary Information

## Figures and Tables

**Table 1:** Nephrotoxicity prediction performances of HPTC and HPTC-like cells estimated using cross validation.

IL-6_max_ & IL-8_max_			
	Sensitivity_CV_ (training/test, %)	Specificity_CV_ (training/test, %)	Balanced accuracy_CV_ (training/test, %)
HPTC1	100.0/92.5	100.0/91.5	100.0/92.0
HPTC2	99.9/88.5	99.6/68.0	99.8/78.3
HPTC3	99.8/87.0	99.8/64.5	99.8/75.8
Mean	**99.9/89.3**	99.8/74.7	99.9/82.0
HPTC-like	99.7/89.0	**100.0/85.0**	**99.8/87.0**

We estimated the training and test performances of random forest classifiers using a standard 10-fold cross validation (CV) procedure with 10 random trials. The mean values shown are the averages of three batches of HPTC (HPTC1, 2, and 3) from different donors (bold = higher values between HPTC-like cells and HPTC).

**Table 2:** Drug-induced nuclear translocation of NF-κB p65 and generation of γH2AX and 4-HNE.

Concentrations	0 μg/ml	1 μg/ml	10 μg/ml	100 μg/ml	1000 μg/ml
	NF-κB p65 - Cytoplasmic/Nuclear Intensity Ratio				
Acarbose	1.43 ± 0.22	1.13 ± 0.02	1.30 ± 0.14	1.39 ± 0.08	1.31 ± 0.19
Ethylene glycol	1.43 ± 0.22	1.78 ± 0.19	1.23 ± 0.10	1.23 ± 0.07	1.36 ± 0.05
Aristolochic Acid	1.30 ± 0.20	1.13 ± 0.08	1.29 ± 0.09	1.21 ± 0.06	**0.78 **±** 0.12**
Cisplatin	1.30 ± 0.20	1.34 ± 0.12	1.34 ± 0.11	**0.83 **±** 0.16**	**0.88 **±** 0.02**
	γH2AX - Log_2_ Average Intensity				
Acarbose	0.00 ± 0.11	0.33 ± 0.27	0.28 ± 0.24	−0.20 ± 0.04	0.13 ± 0.29
Ethylene glycol	0.00 ± 0.11	0.23 ± 0.19	0.15 ± 0.08	−0.14 ± 0.11	0.03 ± 0.13
Aristolochic Acid	−0.02 ± 0.30	0.20 ± 0.24	0.06 ± 0.13	0.53 ± 0.26	**1.57 **± 0.33*
Cisplatin	−0.02 ± 0.30	−0.03 ± 0.14	−0.07 ± 0.27	**0.13 **± 0.04*	**0.82 **± 0.02*
	4-HNE - Log_2_ Average Intensity				
Acarbose	0.02 ± 0.05	−0.01 ± 0.09	−0.18 ± 0.16	0.07 ± 0.07	0.10 ± 0.07
Ethylene glycol	0.02 ± 0.05	−0.05 ± 0.07	−0.07 ± 0.14	0.17 ± 0.03	0.14 ± 0.17
Aristolochic Acid	0.00 ± 0.14	−0.39 ± 0.17	−0.35 ± 0.19	−0.01 ± 0.20	**0.48 **± 0.05*
Cisplatin	0.00 ± 0.14	−0.30 ± 0.21	−0.31 ± 0.18	0.09 ± 0.25	**1.99 **± 0.32*

hiPSC-derived HPTC-like cells were treated with acarbose, ethylene glycol, aristolochic acid and cisplatin at the indicated concentrations of 1, 10, 100 and 1000 μg/ml. 0 μg/ml denotes the vehicle controls without any drugs and two different vehicle controls were used depending on whether drugs were dissolved in water or DMSO. The values show the mean +/− standard deviation (n = 3). In case of NF-κB p65 the cytoplasmic/nuclear intensity ratio was measured. Values <1 indicated a higher fluorescence intensity in the nucleus (as compared to the cytoplasm) and respective samples were considered as being positive for nuclear translocation of NF-κB p65 (bold). In case of γH2AX and 4-HNE the log_2_ average fluorescence intensity values are displayed (with respect to γH2AX the nuclear fluorescence intensities were measured). Significant increases (P <0.05) are indicated by asterisks and are highlighted in bold.
