# Supplementary material for: Prediction of drug-induced nephrotoxicity and injury mechanisms with human induced pluripotent stem cell-derived cells and machine learning methods
Source: Sci Rep. 2015 Jul 27;5:12337. doi: 10.1038/srep12337 (PMC4515747; doi:10.1038/srep12337)
Supplement: Supplementary Information [file srep12337-s1.pdf]

Supplementary Information

Prediction of drug-induced nephrotoxicity and injury mechanisms with human induced pluripotent stem cell-derived cells and machine learning methods

Karthikeyan Kandasamy [1, a], Jacqueline Kai Chin Chuah [1, a], Ran Su [2], Peng Huang [1], Kim Guan Eng [1], Sijing Xiong [1],Yao Li [1], Chun Siang Chia [1], Lit-Hsin Loo [2, 3] and Daniele Zink [1*]

[1] Institute of Bioengineering and Nanotechnology, 31 Biopolis Way, The Nanos, Singapore 138669, Singapore

[2] Bioinformatics Institute, 30 Biopolis Street, #07-01 Matrix, Singapore 138671, Singapore

[3] Department of Pharmacology, Yong Loo Lin School of Medicine, National University of Singapore, 10 Medical Drive, Singapore 117597, Singapore

[a] Both authors contributed equally

* Corresponding author:

Phone: +65 6824 7107, Fax: +65 6478 9080, E-mail addresses: dzink@ibn.a-star.edu.sg

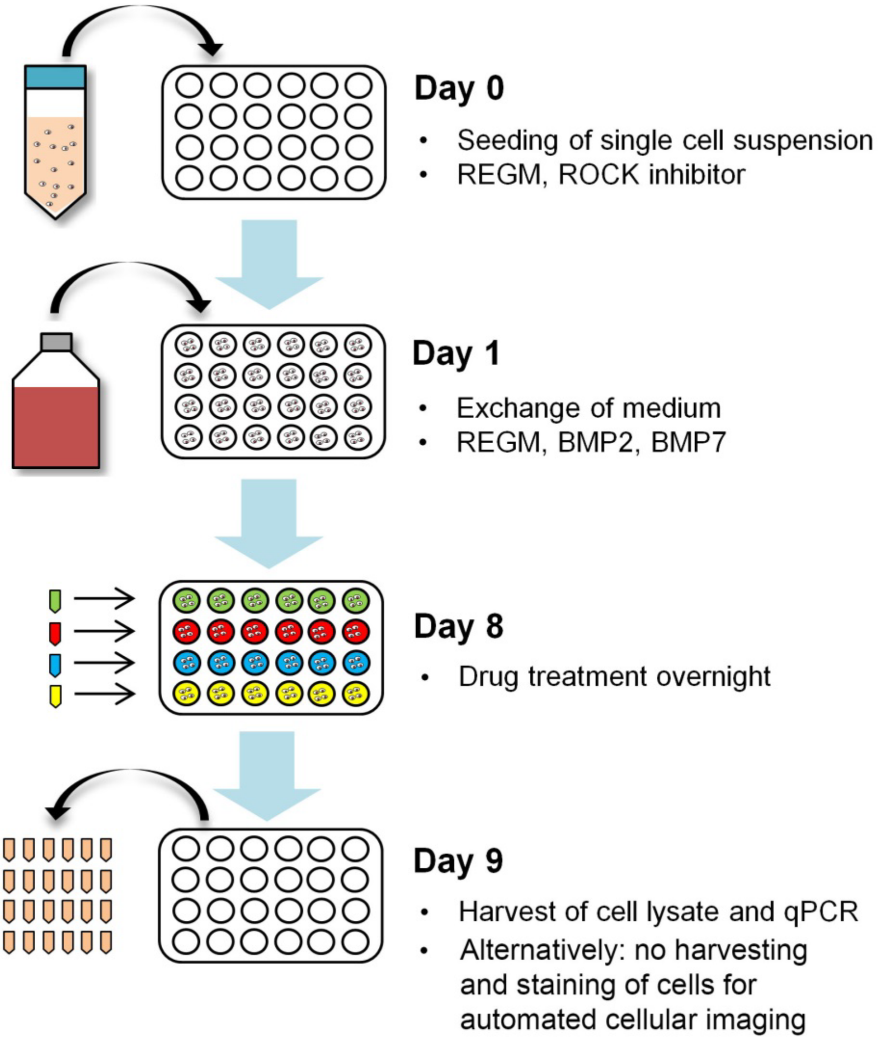

Figure S1: Flow chart of the differentiation procedure with subsequent drug testing. hiPSC are seeded into multi-well plates on day 0. On day 1 the differentiation medium containing BMP2 and BMP7 is added. The cells differentiate during the following week into HPTC-like cells and can be used for drug testing in the evening of day 8. After drug exposure over night the cell lysates are harvested in the morning of day 9 for determining IL6 and IL8 levels by qPCR. Alternatively, cells can be fixed and stained in the morning of day 9 in the micro-well plate used for cell differentiation and used for analysis with automated cellular imaging.

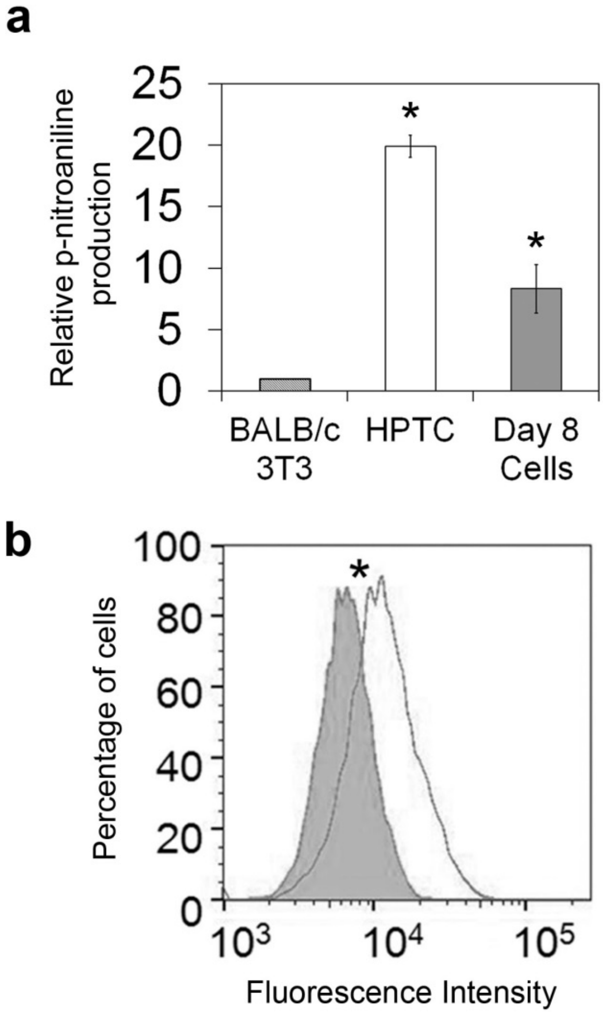

Figure S2: GGT activity and OCT2 activity of iPS(Foreskin)-4-derived d8 cells. a) Relative GGT activity was determined in BALB/c 3T3 fibroblasts (negative control), HPTC (positive control) and iPSC(Foreskin)-4-derived d8 cells. The bars show the mean +/- standard deviation (s.d., n = 3). All results were normalized to the results obtained with BALB/c 3T3 cells, which were set to 1. GGT activity was significantly higher in HPTC and hiPSC-derived d8 cells in comparison to BALB/c 3T3 cells (asterisks) b) To determine OCT2 activity iPS(Foreskin)-4-derived d8 cells were incubated with vehicle (DMSO)-containing cell culture medium, or with cell culture medium containing 50 μm of the OCT2 inhibitor tetrapentylammonium. After incubation for 5 minutes, 25 μm of the fluorescent OCT2 substrate ASP^+ (4-(4-(dimethylamino)styryl)-N-methylpyridinium iodide) were added. After

30 minutes propidium iodide was added for monitoring cell viability and the cells were analysed by FACS. The diagram shows the cellular fluorescence intensities of inhibitor-treated samples (gray) and vehicle controls (white). The inhibitor-treated cells displayed decreased fluorescence intensity, consistent with decreased uptake of the fluorescent OCT2 substrate in the presence of the OCT2 inhibitor. Data analysis yielded an MDR activity factor (MAF) value > 25. Samples with MAF values >25 are considered as being positive for transporter activity (asterisk).

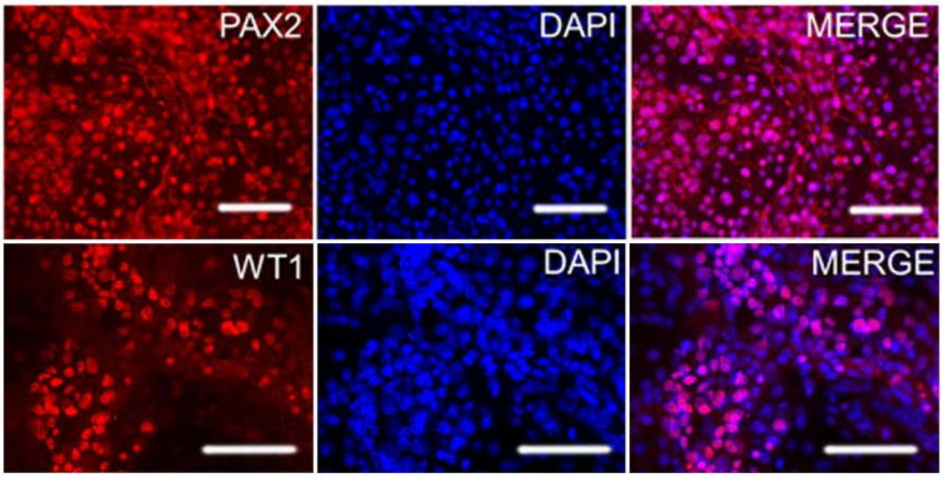

Figure S3: Marker expresson in hiPSC-derived cells. PAX2 and WT1 were detected by immunofluorescence (red; left-hand panels) in iPS(Foreskin)-4-derived d8 cells. 4',6-diamidino-2-phenylindole (DAPI)-stained cell nuclei are shown in blue (middle). The right-hand panels show the merges. Scale bars: 200 µm

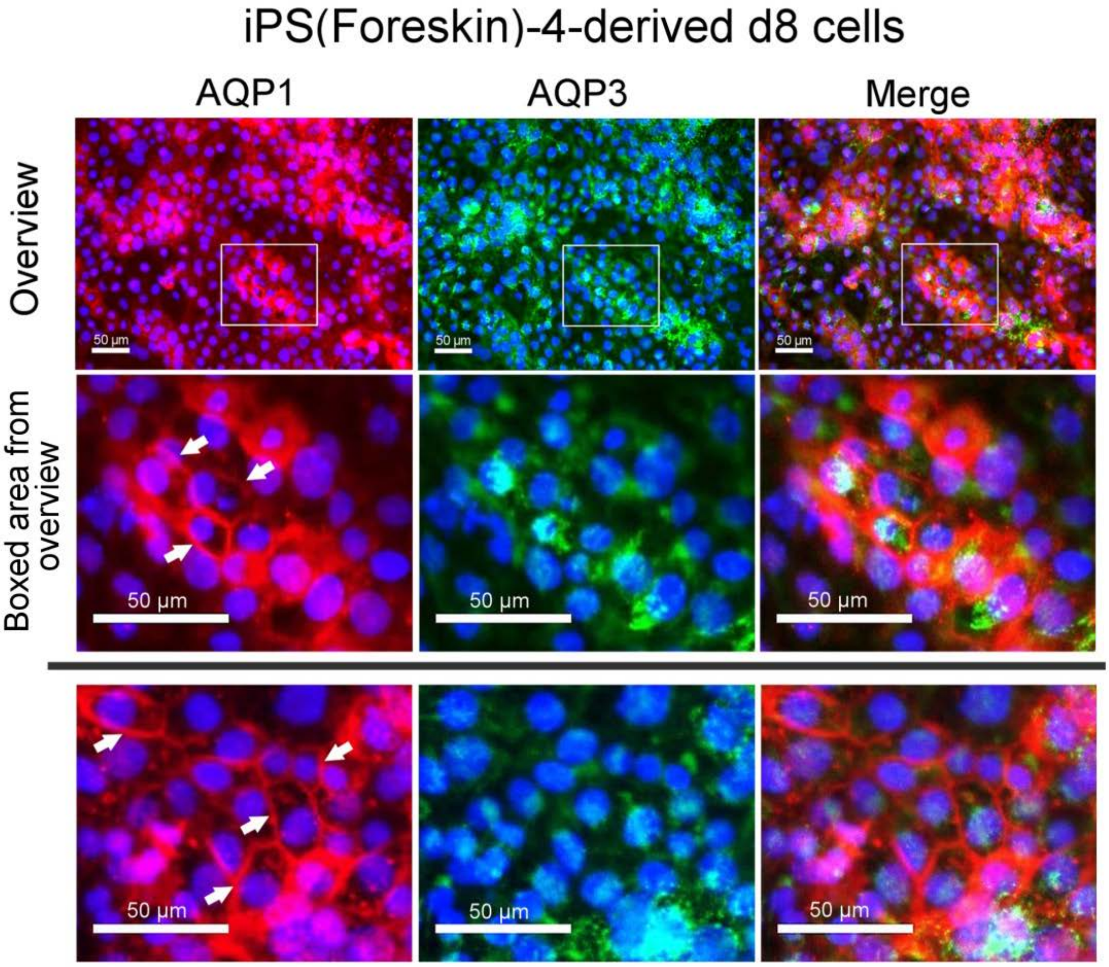

Figure S4: Co-expression of AQP3 and AQP1. AQP1 (red) and AQP3 (green) were detected by co-immunofluorescence in iPS(Foreskin)-4-derived d8 cells. Cell nuclei are shown in blue. The panels show either the AQP1-specific fluorescence (left) or the AQP3-specific fluorescence (middle) or the merges (right). AQP3 is a collecting duct marker, whereas AQP1 expression is specific for proximal tubules. Most iPS(Foreskin)-4-derived d8 cells co-expressed both markers, as seen on the overview images (top) as well as on enlargements of different areas (middle and bottom). Whereas AQP1 shows enrichment at cell surfaces (some surface enrichments highlighted with white arrows; for enrichment of AQP1 at cell surfaces see also Supplementary Fig. S8), AQP3 does not show enrichment at cell surfaces. The immunofluorescence intensity is not uniform for all areas on an image as not all areas localized in the focal plane due to dome formation (compare Fig. 1 c-e). Scale bars: 50 µm.

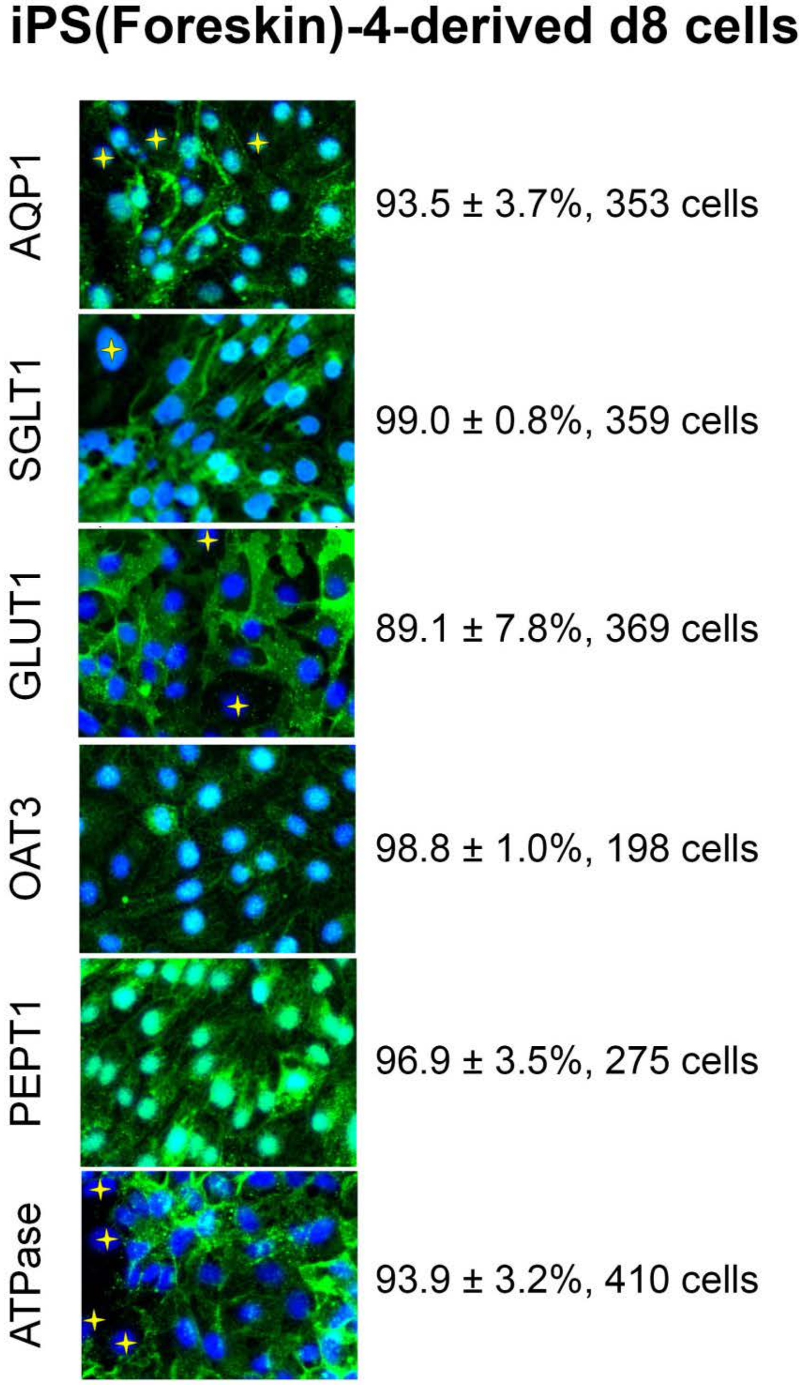

Figure S5: Characterization of iPS(Foreskin)-4-derived d8 cells by immunostaining and image analysis. The panels show epithelia of d8 cells derived from iPS(Foreskin)-4 cells. The PTC-specific markers indicated on the left were detected using immunofluorescence (green: markers; blue: nuclei). Cell numbers and the numbers of cells that were positive or negative for a given marker were quantified by image analysis (see Methods). For each marker at least 3 images were analyzed and the numbers of analyzed cells are indicated on the right, as well

as the average percentages (+/- s. d.) of positive cells. The images illustrate the results (yellow stars: negative cells). These immunofluorescence-based results were consistent with the FACS results (Fig. 2), which showed that in most cases at least ~ 90% of the cells were positive.

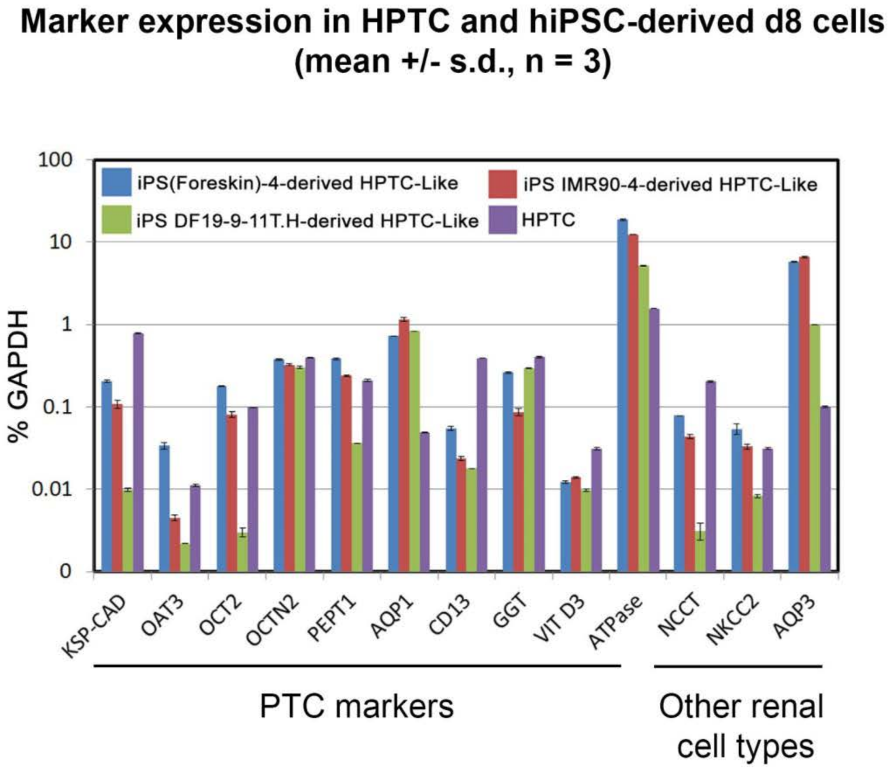

Figure S6: Marker expression determined by qPCR in d8 cells derived from three different hiPSC lines. Expression of the 10 indicated PTC markers and of 3 markers specific for other renal cell types was determined by qPCR (expression levels displayed as % of GAPDH expression). Blue, red and green bars show the results (mean +/- s.d., n = 3) obtained with d8 cells derived from three different hiPSC lines as indicated. Violet bars show the results obtained with HPTC for comparison. The results on iPS(Foreskin)-4-derived cells and HPTC are identical with the results displayed in Fig. 1 b (except OAT3/HPTC) and are shown here again for comparison.

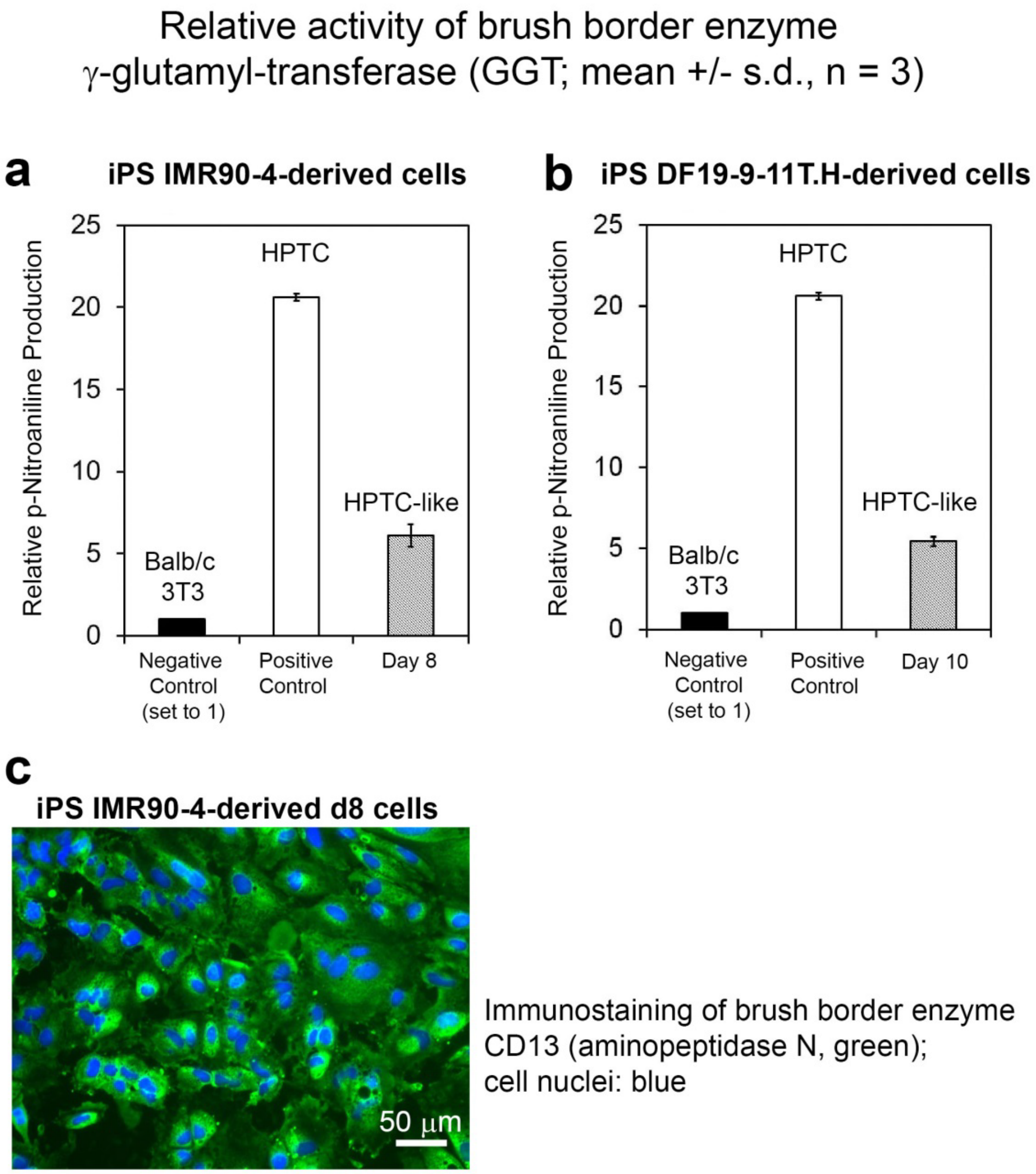

Figure S7: Brush border enzymes in iPS IMR90-4- and iPS DF19-9-11T.H-derived cells. Panels a) and b) show the relative GGT activity as measured by p-nitroaniline production, which was determined in BALB/c 3T3 fibroblasts (negative control), HPTC (positive control) and hiPSC-derived HPTC-like cells. hiPSC-derived cells were tested on day 8 (a) or day 10 (b) of differentiation. The bars show the mean +/- s.d. (n = 3). All results were normalized to the results obtained with BALB/c 3T3 cells, which were set to 1. c) CD13 was detected by immunostaining (green) in iPS IMR90-4-derived d8 cells (cell nuclei: blue). Scale bar: 50 μm.

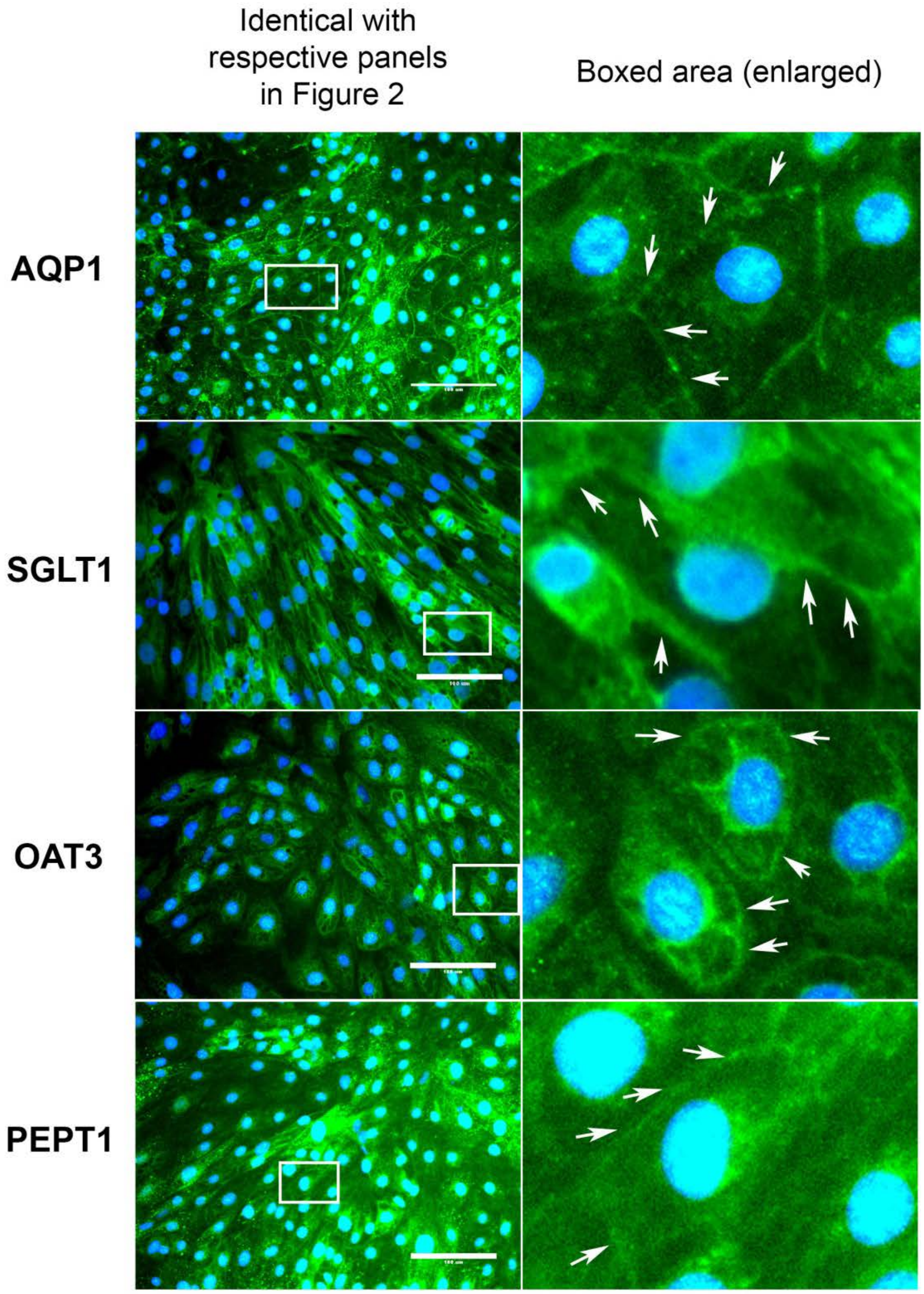

Figure S8: Enrichment of marker proteins at cell surfaces. The left-hand panels show immunostaining of the marker proteins (green) indicated on the left (cell nuclei: blue). The panels are identical with the respective panels shown in the main Fig. 2. The boxed areas are shown enlarged in the right-hand panels. All marker proteins show enrichment at cell surfaces and white arrows point to some of the areas where this is discernible. Scale bars: 100 μm.

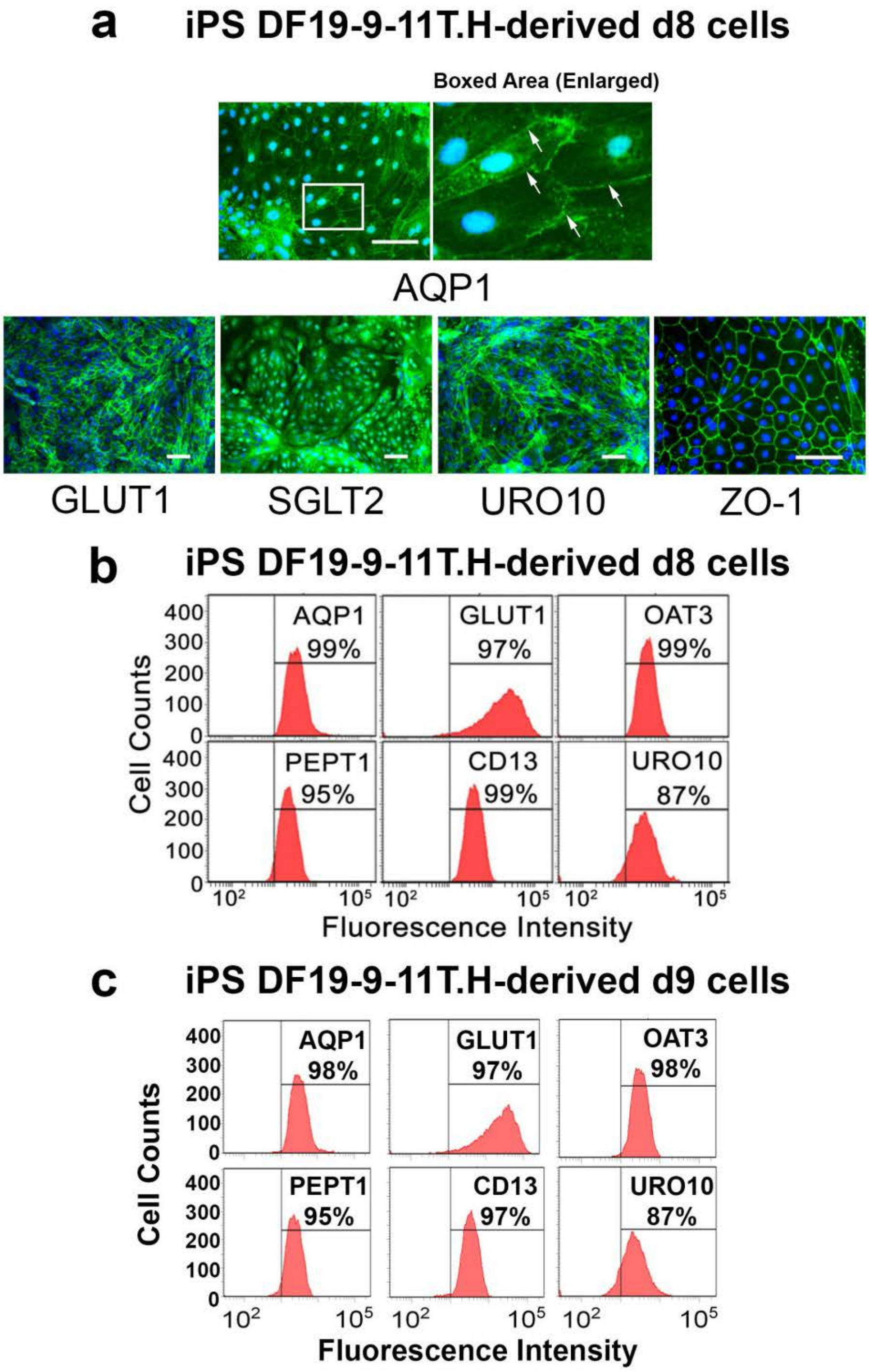

Figure S9: Marker protein expression of iPS DF19-9-11T.H-derived cells. a) Immunostaining results obtained with iPS DF19-9-11T.H-derived d8 cells are shown. The markers detected by immunofluorescence (green) are indicated below the panels (cell nuclei: blue). In case of AQP1 part of the overview image (boxed on left-hand panel) was enlarged (right-hand panel) to show enrichment of the marker at cell surfaces. White arrows point to

some of the areas where enrichment at cell surfaces is discernible. Scale bars: 100 μm. b) FACS results obtained with iPS DF19-9-11T.H-derived d8 cells. The percentages of cells positive for the markers indicated are displayed. c) FACS results obtained with iPS DF19-9-11T.H-derived d9 cells. The percentages of cells positive for the markers indicated are displayed.

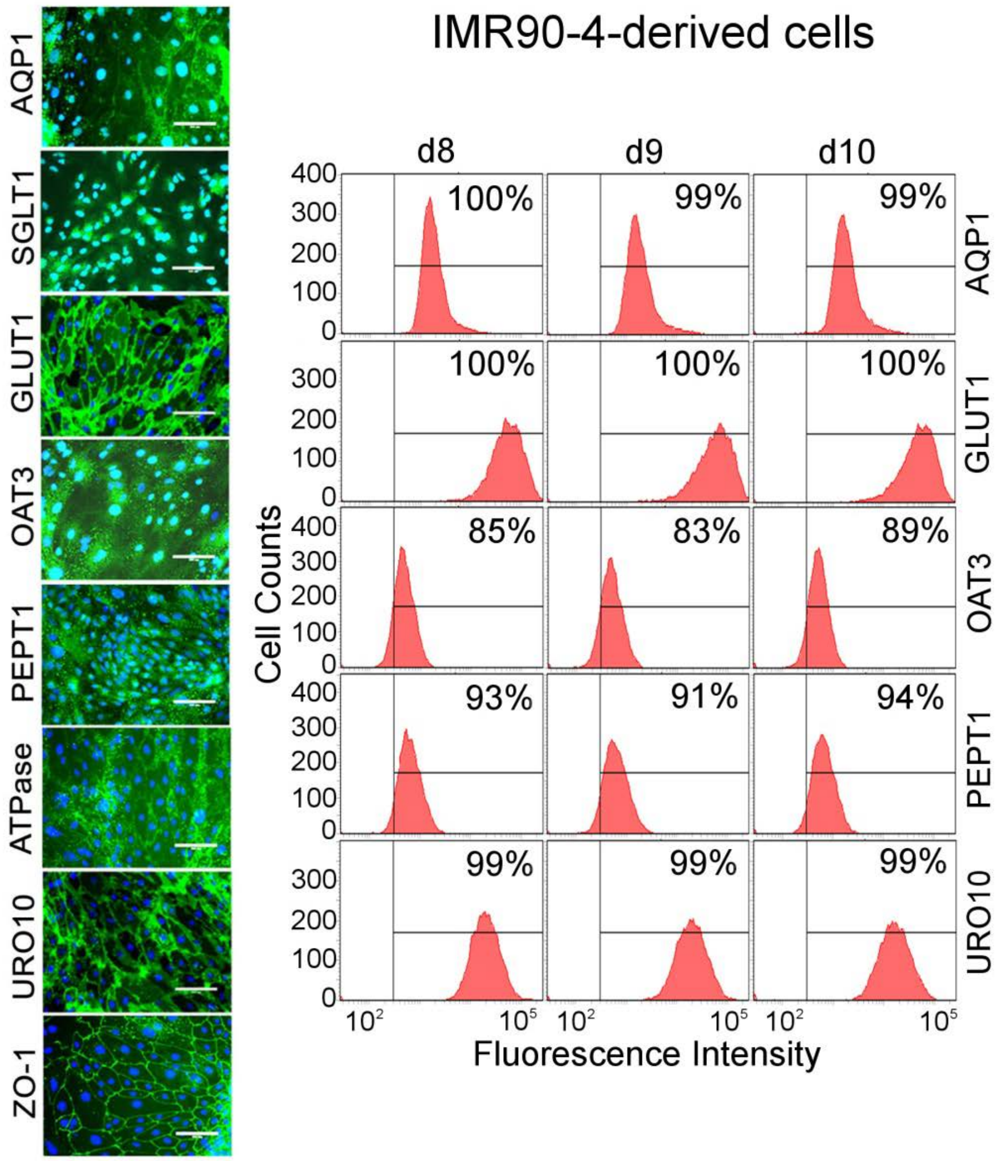

Figure S10: Characterization of iPS IMR90-4-derived cells by immunostaining and FACS. The left-hand panels show epithelia of d8 cells derived from iPS IMR90-4 cells. The PTC-specific markers indicated on the left were detected by immunofluorescence (green: markers, blue: nuclei): Scale bars: 100 μm. The right-hand panels show FACS results obtained with iPS IMR90-4-derived cells harvested on day 8, day 9 and day 10. The percentages of cells positive for the markers indicated on the right are displayed.

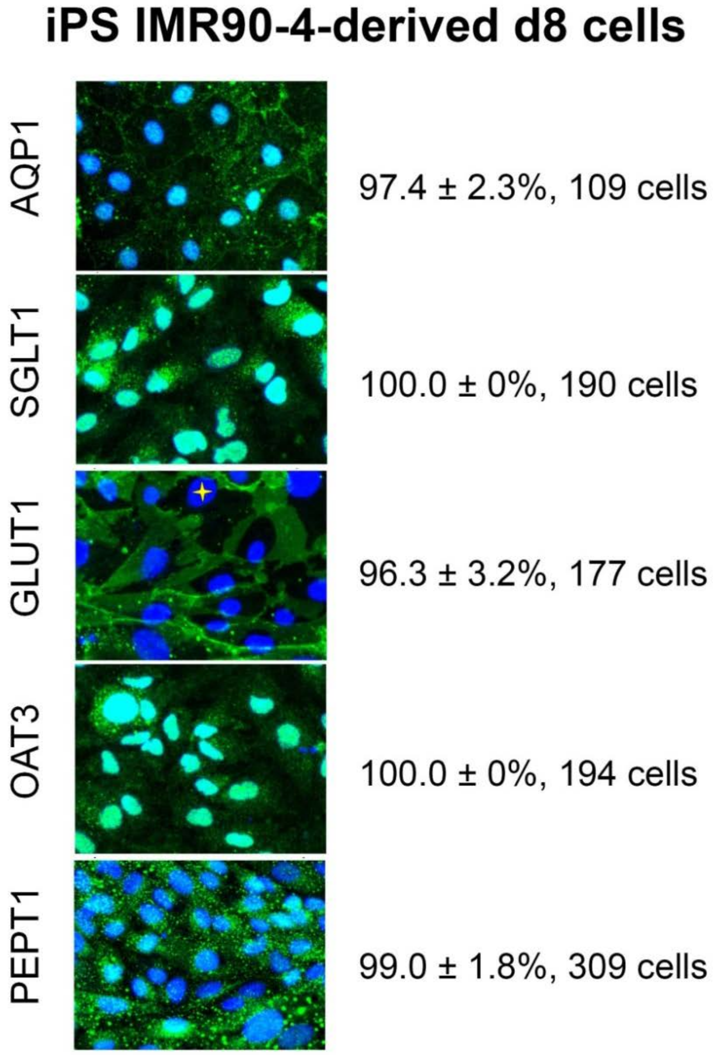

Figure S11: Characterization of iPS IMR90-4-derived d8 cells by immunostaining and image analysis. The panels show epithelia of d8 cells derived from iPS IMR90-4 cells. The PTC-specific markers indicated on the left were detected using immunofluorescence (green: markers; blue: nuclei). Cell numbers and the numbers of cells that were positive or negative for a given marker were quantified by image analysis (see Methods). For each marker at least 3 images were analyzed and the numbers of analyzed cells are indicated on the right, as well as the average percentages (+/- s. d.) of positive cells. The images illustrate the results (yellow stars: negative cells).

**Acronym / Gene ID Description**	**Primer Pairs**	**Amplicon (bp)**
AQP1 / AQP1<br>Aquaporin 1	F 5’-AAGCTCTTCTGGAGGGCAGT-3’<br>R 5’-CACCTTCACGTTGTCCTGGACCG-3’	137
AQP3 / AQP3<br>Aquaporin 3	F 5’-GACGCTGGGAGCCTTCTTG-3’<br>R 5’-GCTGGTTGTCGGCGAAGT-3’	80
ATPase / ATP1B1<br>ATPase, Na+/K+ transporting, beta 1 polypeptide	F 5’-GCTGACCCGCCATCGCCAT-3’<br>R 5’-ACCAACTGCCACCGGTCCTG-3’	114
CD13 / ANPEP<br>Alanyl (membrane) aminopeptidase	F 5’-CACACACCGTTCCTGGATCTCCTCT-3’<br>R 5’-GCTCCAACAGGCGAAGGTCACT-3’	76
DNMT3B / DNMT3B<br>DNA(cytosine-5-)-methyltransferase 3 beta	F 5’-AGTCCTCAAAGAGTTGGGCATAAA-3’<br>R 5’-ACGGTTCCAACAGCAATGG-3’	80
E-CAD / CDH1<br>E-cadherin	F 5’-GAGGACCAGGACTTTGACTT-3’<br>R 5’-AGATACCGGGGGACACTCAT -3’	107
GAPDH / GAPDH<br>Glyceraldehyde-3-phosphate dehydrogenase	F 5’-ACCCCTTCATTGACCTCAACTACA-3’<br>R 5’-CTTGACGGTGCCATGGAATT-3’	80
GDNF / GDNF<br>Glial cell derived neurotrophic factor	F 5’-ACTTGGGTCTGGGCTATGAAAC-3’<br>R 5’-TCGTACGTTGTCTCAGCTGCAT-3’	85
GGT / GGT1<br>Gamma-glutamyl transferase 1	F 5’-TGAGCCCAGAAGTGAGAGCAGTTG-3’<br>R 5’-ATGTCCACCAGCTCAGAGAGGGT-3’	85
GLUT5 / SLC2A5<br>Solute carrier family 2 (facilitated glucose /fructose transporter), member 5	F 5’-CCCCAGCTCTTCATCACTGTTGGC-3’<br>R 5’-TTTGGAACACAAGGAGGGGGCC-3’	148
HOXD11 / HOXD11<br>Homeobox D11	F 5’-AAAAAGCGCTGTCCCTATACCA-3’<br>R 5’-TGAGGTTGAGCATCCGAGAGA-3’	115
IL6 / IL6<br>Interleukin 6	F 5’-TGGCTGCAGGACATGACAAC-3’<br>R 5’-TGAGGTGCCCATGCTACATTT-3’	100
IL8 / IL8<br>Interleukin 8	F 5’-TTGGCAGCCTTCCTGATTTCT-3’<br>R 5’-GGGTGGAAAGGTTTGGAGTATG-3’	110
KIM1 / HAVCR1<br>Hepatitis A virus cellular receptor 1	F 5’-CAGGCTGATCCCATAATGCA-3’<br>R 5’-CTGCCTCTCCACCAACCTTTAC-3’	100
KSP-CAD / CDH16	F 5’-TCCCATGCCTACCTCACCTT-3’	125

KSP-cadherin	R 5'-TTGCAGCGACACACGATCA-3'	
MDR1 / ABCB1 ATP-binding cassette, sub-family B (MDR/TAP), member 1	F 5'-GCCCTTGTTAGACAGCCTCATATTT-3' R 5'-GGACAGGCGGTGAGCAAT-3'	141
MEG / LRP2 Low density lipoprotein receptor-related protein 2 (Megalin)	F 5'-AGACTGGTTCTAACGCCTGTAATC-3' R 5'-GCTCTGTGGGTGGTTCATTGG-3'	171
NANOG / NANOG Nanog homeobox	F 5'-TCGCAAAAAAGGAAGACAAGGT-3' R 5'-GAGTACACACAGCTGGGTGGAA-3'	80
NBC1 / SLC4A4 Solute carrier family 4 (sodium bicarbonate cotransporter), member 4	F 5'-CCAAACTGGAGGAGCGACGGAAG-3' R 5'-CACACACATGCTTGAGGAAGGA -3'	90
N-CAD / CDH2 N-cadherin	F 5'-CCCATACACCAGCCTGGAACGC-3' R 5'-TGGGTCGGTCTGGATGGCGA-3'	80
NCCT / SLC12A3 Thiazide-sensitive sodium chloride cotransporter	F 5'-CACCAAGAGGTTTGAGGACATG-3' R 5'-GACAGTGGCCTCATCCTTGAA-3'	70
NGAL / LCN2 Lipocalin 2	F 5'-CAAGGAGCTGACTTCGGAACTAA-3' R 5'-TGCACTCAGCCGTCGATACA-3'	120
NKCC2 / SLC12A1 Solute carrier family 12 (sodium/potassium/chloride transporter), member 1	F 5'-TGGGGAGTCATGCTCTTCATTCGC-3' R 5'-CCACGAACAAACCCGTTAGTTGC-3'	149
OAT1 / SLC22A6 Solute carrier family 22 (organic anion transporter), member 6	F 5'-TCTACTCCTGGTTCTTCATTG-3' R 5'-CGGAGTACCTCCATACTCAAT-3'	142
OAT3 / SLC22A8 Solute carrier family 22 (organic anion transporter), member 8	F 5'-GCCCTTGGACTTGCAGACCG-3' R 5'-ACCTGTTTGCCTGATGACTG-3'	127
OCT2 / SLC22A2 Solute carrier family 22 (organic cation transporter), member 2	F 5'-GCTGTACCCCACATTCATTAGGA-3' R 5'-GGGAGCTCAAGCCAGATGTTA-3'	120

OCT3/4 / POU5F1<br>POU class 5 homeobox 1	F 5’-GGAGGAAGCTGACAACAATGAAA-3’<br>R 5’-GGTTGCCTCTCACTCGGTTCT-3’	110
OCTN2 / SLC22A5<br>Solute carrier family 22 (organic cation/carnitine transporter), member 5	F 5’-GGTTTGGCCGGAAGAATGT-3’<br>R 5’-CCATGCCTACAAGGACAAACAG-3’	120
OSR1 / OSR1<br>Odd-skipped related transciption factor 1	F 5’-CCCTGCAGCTCACCAACTACT-3’<br>R 5’-AGATGGTCCGAAGGCACTGT-3’	70
PAX2 / PAX2<br>Paired box 2	F 5’-CTTCCAGGCATCAGAGCACAT-3’<br>R 5’-GTGGATGCAGATAGACTCGACTTG-3’	105
PEPT1 / SLC15A1<br>Solute carrier family 15 (oligopeptide transporter), member 1	F 5’-CAGTGGGCCGAGTACATTCTATT-3’<br>R 5’-TCTCCGCTGGGTTGATGTAAG-3’	100
PODXL / PODXL<br>Podocalyxin-like	F 5’-ACCTACCCTGCCAGAGACCAT-3’<br>R 5’-AGATCCTCACACTTTGCCCAGTT-3’	120
PPIA / PPIA<br>Peptidylprolyl isomerase A (cyclophilin A)	F 5’-GTGCATGCCTAGTCCTAGCTGAT-3’<br>R 5’-CTCACTCTAGGCTCAAGCAATCC-3’	64
SGLT2 / SLC5A2<br>Solute carrier family 5 (sodium/glucose cotransporter), member 2	F 5’-ACGCCTGATTCCCGAGTTCT-3’<br>R 5’-AGAACAGCACAATGGCGAAGT-3’	110
SIX2 / SIX2<br>SIX homeobox 2	F 5’-AGGAAAGGGAGAACAACGAGAA-3’<br>R 5’-GAGCTGCCTAACACCGACTTG-3’	82
SLC34A1 / SLC34A1<br>Solute Carrier Family 34 (Type II Sodium/Phosphate Contransporter), Member 1	F 5’-CTGGGTCACAGGCTACTTTGC-3’<br>R 5’-GCCCTCTCAATGCTGATCACA-3’	126
SMA / ACTA2<br>Actin, alpha 2, smooth muscle, aorta	F 5’-TCATCACCAACTGGGACGAC-3’<br>R 5’-ATGCTCTTCAGGGGCAACAC-3’	80
SOX2 / SOX2<br>SRY (sex determining region Y)-box 2	F 5’-ATCCCATCACCCACAGCAA-3’<br>R 5’-GTCGGCATCGCGGTTTT-3’	81
T / T<br>Brachyury	F 5’-GGGTCCACAGCGCATGAT-3’<br>R 5’-TTTAAGAGCTGTGATCTCCTCGTT-3’	95

UMOD / UMOD<br>Uromodulin	F 5’-TGGCTTCAGGACACCAGACATCAG-3’<br>R 5’-AGCACCTGCCCAAAGGAAAGACG-3’	77
VIM / VIM<br>Vimentin	F 5’-ACCTGAGGGAAACTAATCTG-3’<br>R 5’-CGTTGATAACCTGTCCATCT-3’	105
VIT D3 / CYP27B1<br>Cytochrome P450, family 27, subfamily B, polypeptide 1 (synthesizes 1alpha,25-dihydroxyvitamin D3)	F 5’-GGAAATTCTCGTGTCCCAGA-3’<br>R 5’-TGACACAGAGTGACCAGCGTA-3’	80
WT1 / WT1<br>Wilms tumor 1	F 5’-AACAGCAACAGCAAGAAATAAATCA-3’<br>R 5’-GACCTCGGGAATGTTAGACAAGAT-3’	71
ZO-1 / TJP1<br>Tight junction protein 1	F 5’-GAGAGGATTTGTCCGCTCAG-3’<br>R 5’-AGGCCTCAGAAATCCAGCTT-3’	86

Table S1: Details of markers used for qPCR. The table lists all markers used for qPCR. The acronyms used by us are alphabetically listed. Gene IDs and descriptions follow the nomenclature of the HUGO Gene Nomenclature Committee (HGNC) (http://www.genenames.org/). The primer pairs used for qPCR (F: forward, R: reverse) and amplicon sizes in base pairs (bp) are provided.

	Compound	IL6 Expression Levels				
		0	1	10	100	1,000
1	Aristolochic acid	1.0 ± 0.0	3.9 ± 0.5	3.3 ± 0.2	7.3 ± 1.6	9.3 ± 0.5
2	Arsenic (III) oxide	1.0 ± 0.1	1.5 ± 0.1	5.2 ± 0.1	14.2 ± 2.1	8.2 ± 0.3
3	Bismuth (III) oxide	1.0 ± 0.2	1.2 ± 0.1	1.3 ± 0.2	2.1 ± 0.1	3.6 ± 0.3
4	Cadmium chloride	1.1 ± 0.1	3.6 ± 0.4	10.9 ± 0.4	10.3 ± 1.6	11.8 ± 0.5
5	Cephalosporin C	1.0 ± 0.0	1.6 ± 0.2	1.7 ± 0.2	9.8 ± 2.2	24.3 ± 1.6
6	Cisplatin	1.0 ± 0.1	6.0 ± 0.8	5.5 ± 1.0	3.9 ± 0.3	ND
7	Citrinin	1.0 ± 0.1	4.1 ± 0.1	9.2 ± 0.3	24.5 ± 3.0	5.4 ± 0.1
8	Copper (II) chloride	1.0 ± 0.1	1.2 ± 0.1	2.2 ± 0.2	22.5 ± 2.2	13.4 ± 0.0
9	5-Fluorouracil	1.0 ± 0.0	6.2 ± 0.7	6.4 ± 0.3	9.2 ± 1.2	1.1 ± 0.2
10	Gentamicin	1.0 ± 0.1	1.6 ± 0.3	1.7 ± 0.3	2.3 ± 0.3	12.1 ± 0.4
11	Gold (I) chloride	1.0 ± 0.1	1.2 ± 0.2	1.2 ± 0.1	2.9 ± 0.2	23.3 ± 0.4
12	Lead acetate	1.0 ± 0.1	1.3 ± 0.3	1.2 ± 0.3	1.5 ± 0.1	4.3 ± 0.8
13	Paraquat	1.0 ± 0.0	1.6 ± 0.2	1.8 ± 0.2	5.4 ± 0.6	3.5 ± 0.3
14	Potassium dichromate	1.0 ± 0.1	1.2 ± 0.4	1.0 ± 0.0	3.3 ± 0.3	3.7 ± 0.4
15	Puromycin	1.0 ± 0.1	1.9 ± 0.2	14.1 ± 1.2	29.0 ± 1.2	26.6 ± 4.3
16	Rifampicin	1.0 ± 0.1	5.8 ± 0.9	4.5 ± 0.3	2.3 ± 0.2	9.3 ± 0.6
17	Tacrolimus	1.0 ± 0.1	6.6 ± 0.6	6.1 ± 0.2	ND	ND
18	Tobramycin	1.0 ± 0.1	1.3 ± 0.1	1.3 ± 0.1	1.4 ± 0.1	2.7 ± 0.4
19	Acetaminophen	1.0 ± 0.1	1.3 ± 0.0	1.2 ± 0.1	1.4 ± 0.2	0.3 ± 0.1
20	Ethylene glycol	1.0 ± 0.1	1.2 ± 0.1	1.1 ± 0.2	1.2 ± 0.1	1.6 ± 0.2
21	Lincomycin	1.0 ± 0.0	1.2 ± 0.1	1.3 ± 0.1	1.2 ± 0.1	1.4 ± 0.2
22	Lindane	1.0 ± 0.0	7.3 ± 1.7	6.4 ± 0.5	5.1 ± 0.4	0.8 ± 0.0
23	Lithium chloride	1.0 ± 0.1	1.2 ± 0.0	1.1 ± 0.1	1.2 ± 0.1	1.8 ± 0.1
24	Phenacetin	1.0 ± 0.0	7.2 ± 0.2	7.0 ± 1.1	5.3 ± 0.1	0.7 ± 0.2
25	Valacyclovir	1.0 ± 0.1	1.3 ± 0.1	1.3 ± 0.1	1.6 ± 0.1	2.8 ± 0.4
26	Vancomycin	1.0 ± 0.1	1.5 ± 0.3	1.4 ± 0.1	1.4 ± 0.1	3.1 ± 0.2
27	Acarbose	1.0 ± 0.0	1.2 ± 0.1	1.5 ± 0.3	1.3 ± 0.2	1.4 ± 0.4
28	Glycine	1.0 ± 0.3	1.6 ± 0.1	1.9 ± 0.3	1.7 ± 0.2	1.9 ± 0.2
29	Melatonin	1.0 ± 0.1	6.5 ± 0.8	6.1 ± 0.4	4.4 ± 0.4	1.1 ± 0.1
30	Ribavirin	1.0 ± 0.1	1.4 ± 0.1	1.5 ± 0.1	1.2 ± 0.3	1.0 ± 0.1

Table S2: Compound-induced IL6 expression in iPS(Foreskin)-4-derived d8 cells. The cells were treated on the evening of d8 with the compounds listed on the left. Group 1 (nephrotoxicants that are directly toxic for PTC) comprised compounds 1-18. Group 2 (compounds that are not toxic for PTC) comprised compounds 19-30. Compounds 19-26 are

nephrotoxicants that do not damage PTC directly and compounds 27-30 are not nephrotoxic in humans. Each compound was applied at concentrations of 1 μg/ml, 10 μg/ml, 100 μg/ml and 1,000 μg/ml. 0 represents the vehicle control (compound concentration: 0 μg/ml). IL6 expression levels were determined by qPCR on the morning of d9. In some cases IL6 expression levels could not be determined (ND) due to massive cell death. The results show the mean +/- s.d. (n = 3). All results were normalized to the respective vehicle controls and the means of the vehicle controls were set to 1.

	Compound	IL8 Expression Levels				
		0	1	10	100	1,000
1	Aristolochic acid	1.0 ± 0.3	5.0 ± 0.8	4.8 ± 0.6	43.5 ± 7.2	1.6 ± 0.9
2	Arsenic (III) oxide	1.0 ± 0.1	1.2 ± 0.5	4.2 ± 0.1	1.5 ± 0.2	3.9 ± 1.6
3	Bismuth (III) oxide	1.0 ± 0.2	0.4 ± 0.1	2.6 ± 0.2	33.0 ± 1.6	192.7 ± 18.4
4	Cadmium chloride	1.1 ± 0.3	15.0 ± 0.8	7.6 ± 0.4	4.4 ± 0.6	20.5 ± 8.3
5	Cephalosporin C	1.0 ± 0.1	1.0 ± 0.1	0.7 ± 0.1	1.5 ± 0.9	ND
6	Cisplatin	1.0 ± 0.0	3.0 ± 1.8	3.3 ± 1.1	5.8 ± 1.0	ND
7	Citrinin	0.9 ± 0.0	2.2 ± 0.2	1.3 ± 0.5	2.5 ± 0.3	ND
8	Copper (II) chloride	1.0 ± 0.2	7.2 ± 0.6	67.1 ± 18.1	58.2 ± 3.2	ND
9	5-Fluorouracil	1.0 ± 0.1	7.4 ± 1.4	9.2 ± 1.4	18.3 ± 2.2	5.7 ± 0.5
10	Gentamicin	1.0 ± 0.2	0.7 ± 0.3	0.6 ± 0.1	2.2 ± 0.5	216.2 ± 16.2
11	Gold (I) chloride	1.0 ± 0.2	0.9 ± 0.2	0.9 ± 0.1	2.0 ± 0.0	1.2 ± 0.2
12	Lead acetate	1.0 ± 0.1	0.4 ± 0.2	1.3 ± 0.1	5.0 ± 0.8	29.4 ± 5.1
13	Paraquat	1.0 ± 0.3	0.7 ± 0.2	1.5 ± 0.3	2.9 ± 0.4	25.5 ± 8.5
14	Potassium dichromate	1.0 ± 0.1	0.5 ± 0.0	0.7 ± 0.1	0.8 ± 0.0	1.2 ± 0.0
15	Puromycin	1.0 ± 0.3	0.8 ± 0.2	255.7 ± 28.2	3,284.1 ± 1,069.3	3,852.3 ± 309.9
16	Rifampicin	1.0 ± 0.1	2.5 ± 0.4	3.4 ± 0.3	9.7 ± 0.3	18.2 ± 1.2
17	Tacrolimus	1.2 ± 0.6	7.8 ± 0.4	5.1 ± 0.6	ND	ND
18	Tobramycin	1.0 ± 0.2	0.5 ± 0.1	0.6 ± 0.1	0.9 ± 0.3	25.5 ± 1.5
19	Acetaminophen	1.0 ± 0.1	0.8 ± 0.3	1.2 ± 0.2	0.5 ± 0.1	0.1 ± 0.0
20	Ethylene glycol	1.1 ± 0.3	0.9 ± 0.0	1.5 ± 0.4	2.0 ± 1.6	1.7 ± 0.4
21	Lincomycin	1.0 ± 0.1	1.2 ± 1.2	1.2 ± 0.3	2.4 ± 0.9	6.1 ± 0.5
22	Lindane	1.0 ± 0.2	27.4 ± 6.2	46.2 ± 22.4	85.0 ± 19.1	4.2 ± 1.3
23	Lithium chloride	1.1 ± 0.3	0.8 ± 0.2	1.2 ± 0.0	1.5 ± 0.1	27.4 ± 2.0
24	Phenacetin	1.1 ± 0.7	23.3 ± 3.1	11.1 ± 0.8	7.7 ± 4.7	6.4 ± 2.1
25	Valacyclovir	1.0 ± 0.2	1.5 ± 0.1	1.4 ± 0.1	1.0 ± 0.5	32.5 ± 1.4
26	Vancomycin	1.0 ± 0.2	0.7 ± 0.0	0.6 ± 0.2	2.9 ± 0.2	98.5 ± 4.5
27	Acarbose	1.0 ± 0.0	0.5 ± 0.1	0.6 ± 0.2	0.4 ± 0.1	1.6 ± 0.2
28	Glycine	1.2 ± 0.5	1.6 ± 0.6	3.1 ± 1.5	2.5 ± 0.6	4.9 ± 1.9
29	Melatonin	1.0 ± 0.1	9.8 ± 2.4	6.9 ± 1.0	1.9 ± 0.9	ND
30	Ribavirin	1.0 ± 0.1	1.1 ± 0.4	0.5 ± 0.1	0.2 ± 0.0	0.2 ± 0.0

Table S3: Compound-induced IL8 expression in iPS(Foreskin)-4-derived d8 cells. For details see legend of Table S2.
